# Assessment of sleep in older people within comprehensive geriatric assessments: a scoping review

**DOI:** 10.1093/ageing/afag094

**Published:** 2026-05-26

**Authors:** Louise D A C Organista, Dayana El Nsouli, Janeme Lam, Manzar Maqsood, Tawfiq Alqeisi, Tiffany K L Feather, Jemima T Collins

**Affiliations:** University Hospitals of Derby and Burton NHS Foundation Trust – Pharmacy, Derby, Derbyshire, UK; School of Health and Care Sciences, University of Lincoln, Lincoln, England, UK; University Hospitals of Derby and Burton NHS Foundation Trust – Pharmacy, Derby, Derbyshire, UK; School of Pharmacy, University of Nottingham, Nottingham, England, UK; Northampton General Hospital NHS Trust – Pharmacy, Northampton, England, UK; University Hospitals of Derby and Burton NHS Foundation Trust – Pharmacy, Derby, Derbyshire, UK; Chesterfield Royal Hospital, Trauma and Orthopaedics, Chesterfield, England, UK; University Hospitals of Derby and Burton NHS Foundation Trust – Pharmacy, Derby, Derbyshire, UK; Academic Unit 3, School of Medicine, University of Nottingham, Nottingham, England, UK; Department of Medicine for the Elderly, University Hospitals of Derby and Burton NHS Foundation Trust, Derby, England, UK

**Keywords:** sleep, CGA, assessment, insomnia, systematic review, older people

## Abstract

**Background:**

Sleep problems are highly prevalent in the older population, with associated physical and mental health effects. They should be identified within a Comprehensive Geriatric Assessment (CGA), a multidimensional evaluation of an older person’s health. However, the extent to which sleep is routinely assessed within CGAs and the methods used, remain unclear.

**Objectives:**

The aim of the review was to describe the assessment tools, practitioners involved and the settings in which sleep assessments were conducted within CGAs.

**Methods:**

A systematic search was conducted across five electronic databases and grey literature sources between January 1993 and June 2025. Relevant data including healthcare setting, practitioners involved, sleep assessment tool(s) and questions used were extracted.

**Results:**

Of 1842 identified references, 136 were included for review. Across 54.4% sources, 28 different assessment tools were identified, with the Pittsburgh Sleep Quality Index being most used (21.3%). General screening questions were utilised in 42.6% sources, most often (81.6%), in place of an assessment tool. The most frequent question related to difficulty falling asleep. Sleep assessments were commonly conducted by nurse/nurse practitioners and geriatricians. They were predominantly performed in secondary care (55.9%), particularly within outpatient geriatric clinics.

**Conclusions:**

Sleep assessment within CGAs varies globally. Incorporating screening questions regarding sleep problems could prompt a more holistic assessment of health and wellbeing in the older person, which could feasibly be performed within a CGA by any practitioner. Guidance defining the standards for assessing sleep within a CGA is needed.

## Key Points

- Sleep complaints are highly prevalent in the older population but are under-recognised.
- Sleep problems should be identified within a Comprehensive Geriatric Assessment using validated tools or screening questions.
- Sleep assessment is possible in any healthcare setting, although outpatient geriatric clinics provide suitable opportunity.
- Sleep can be assessed by any healthcare practitioner in the multidisciplinary team (MDT).
- Guidance defining the standards for assessing sleep within a Comprehensive Geriatric Assessment is needed to inform practice.

## Introduction

Sleep is a fundamental physiological process essential for preserving physical health, cognition and emotional wellbeing in older people [1]. Ageing is associated with poorer sleep quality due to reductions in slow-wave and rapid eye movement sleep, increased nocturnal awakenings and circadian rhythm changes, all of which impair restorative sleep [2]. Up to 75% of older people have sleep problems including insomnia and daytime drowsiness [3, 4]. These issues are associated either directly or bidirectionally with functional decline, falls, polypharmacy, depression, malnutrition and cognitive decline [5–9]. Despite this, up to half of sleep problems are underreported and dismissed as ‘normal’ ageing [10], which may delay diagnosis and contribute to reduced quality of life. Timely sleep assessment therefore presents an important opportunity for early intervention to prevent or mitigate common problems in later life.

The Comprehensive Geriatric Assessment (CGA) is a multidimensional and holistic evaluation of a person’s medical, psychological and functional health [11]. It aims to assess and address an individual’s needs through the development of a coordinated care plan [12]. Incorporating sleep assessment within the CGA may support earlier identification of primary sleep disorders and facilitate coordinated management of underlying psychosocial contributors, such as alcohol dependence. Recognition of sleep disturbance may also crucially aid in diagnosing clinical conditions in which sleep changes are a prominent feature, such as dementia with Lewy bodies [13].

Hypnotic medicines are increasingly used to manage insomnia but have associated risks, such as falls and fractures, particularly in older people [14, 15]. Addressing the complexities of sleep within the broader context of the CGA can therefore support the use of safer, non-pharmacological, lifestyle-based interventions and reduce inappropriate prescribing.

### Objective

Despite recognition that sleep is an essential component of holistic assessment in older people’s health [16], the extent to which it is routinely evaluated within the CGA, and the methods used, remain unclear. This scoping review aimed to describe the tools, methods, practitioners involved and clinical setting for sleep assessment within the CGA in research and practice globally. A clearer understanding of this may allow comparability between services, highlight best practice and identify opportunities for intervention to improve clinical outcomes for older people.

## Methods

### Protocol and registration

The scoping review was conducted according to protocol on Open Science Framework (https://osf.io/pfbc9) published on 18 June 2025. Findings were reported according to the Preferred Reporting Items in Systematic Reviews and Meta-analyses—Scoping Reviews Extension (PRISMA-ScR) [17].

### Eligibility criteria

Older people, mean or median age ≥60 years who had sleep assessed within a CGA, in any healthcare context, was the topic of interest. Where the term CGA was not stated, sources that carried out a multidimensional assessment including physical, functional, psychological, social and environmental factors [11] were included. All research study types were eligible including qualitative, quantitative and mixed research methods published in the English language, due to limited translation resources. Grey literature such as conference abstracts, guidelines and healthcare policies was also included. Literature published from January 1993, following publication of a landmark meta-analysis of controlled trials which demonstrated effectiveness of CGA programmes [18], until June 2025 was searched.

### Information sources and search strategy

Searches were carried out on 19 June 2025 on CINAHL Ultimate, Embase, MEDLINE, PsychInfo® and Web of Science databases. Search concepts ‘comprehensive geriatric assessment,’ ‘older person’ and ‘sleep’ were used. Search terms were reviewed and refined with support of an expert subject librarian. The full search strategy is shown in Supplementary Appendix 1.

The grey literature databases and sources included: Acute Frailty Network, AgeInfo, Age UK, American Geriatrics Society, British Geriatrics Society, The British Library, Canadian Geriatrics Society, Cochrane Library, EThOS, The Health Foundation, Health Innovation East Midlands, The King’s Fund, Midlands Sleep Group, National Voices, National Institute for Health and Care Excellence (NICE), PROSPERO, Royal College of Nursing, Royal College of Physicians, Royal College of Psychiatrists and World Health Organisation (WHO). Google Advanced Search was used to facilitate searching each website as there was a lack of standardised search bar or ability to use Boolean search operators. Additional references were obtained through searching reference lists, citation searches and Google Advanced Search using ‘.gov.uk’ and ‘.nhs.uk’ domains. Search terms used the concepts ‘comprehensive geriatric assessment’ and ‘sleep.’ Final search results were exported into EndNote™ 21 [19] and imported into Covidence™ [20] which removed duplicates.

### Selection of sources of evidence

Title and abstract screening were conducted independently by two reviewers. Sources were included if the concepts of CGA in older people and sleep were stated. Excluded references (Supplementary Appendix 4) were those which involved a younger patient population <60 years, studies not relating to the CGA and sleep assessment without a CGA context. Conflicts were discussed with a third reviewer until consensus was reached. An independent full text review between principal reviewer and a second reviewer proceeded, noting reasons for excluding full texts.

### Data charting process

Data were charted using a template developed in Covidence™ [20] (Supplementary Appendix 2). This was revised in an iterative process, after piloting a range of evidence sources. Data charting was carried out independently in pairs with comparison of data and consensus reached between each, before being exported as a completed table.

### Data items

Charted data were recorded in a table using Microsoft® Excel® Version 2507 under the following headings: author(s), type of source e.g. (primary research/conference abstract/guideline), title of source, year, country, study aim, sleep assessment focus of study (Y/N), patient information (age/comorbidities), exclusion criteria, healthcare setting, practitioner involved in sleep assessment, sleep assessment tool(s) used and questions asked about sleep. This was adapted from the JBI Manual for Evidence Synthesis [21] to include all relevant data to inform the review aims.

### Synthesis of results

A summary table of sleep assessment tools used in the review was produced. Tool outcome measures were classified according to their primary construct. These included: sleep quality/disturbance, daytime sleepiness, sleep-disordered breathing risk, sleep hygiene behaviours and primary sleep disorders. Instruments not designed specifically for sleep were categorised based on the presence of sleep-related items. The topics of commonly used assessment questions were described. Healthcare practitioner involvement was summarised in table format. Healthcare setting, including country involvement, was described. Participant comorbidities and reasons for excluding participants from the study (if applicable) were stated. A PRISMA flow chart was created using Covidence™ [20].

## Results

### Selection of sources of evidence

**Figure 1:**
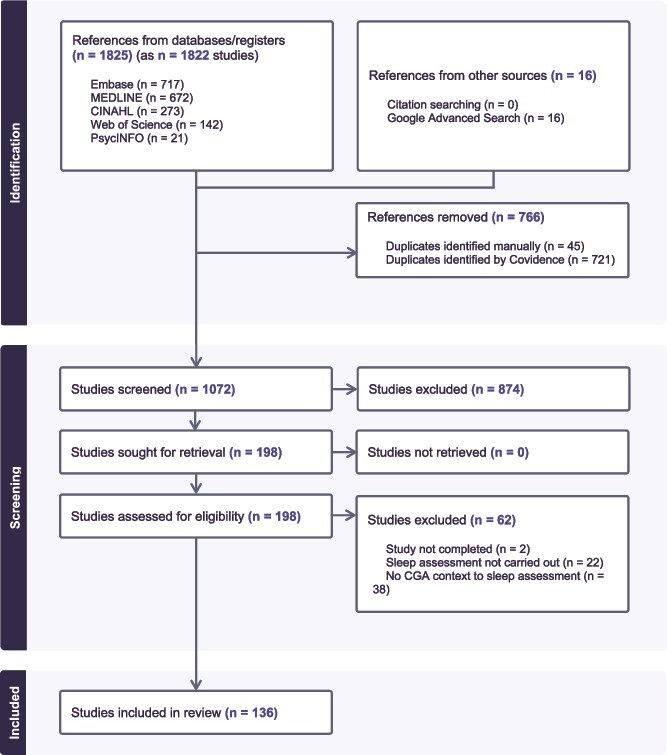
PRISMA flow chart.

From 1825 database and 16 grey literature citations, 136 sources were included (Figure 1). Two pairs of publications were merged due to corrigenda items.

### Characteristics of sources of evidence

Of the 136 sources, 67.6% were primary research studies [9, 22–112] and 32.4% were from grey literature sources [113–156] including conference abstracts, posters and presentations (20.6%), guidelines (10.3%) and clinical trial protocols (1.5%). Mean participant age was 75.5 ± 6.4 years. In 20.6% sources [9, 25, 28–32, 37, 41, 42, 44, 46, 51, 55, 57, 65, 68, 77, 82, 84, 85, 87, 95, 105, 111, 113, 115, 151], assessment of sleep was the main focus of study.

### Sleep assessment within the CGA

Over half (54.4%) of sources used an assessment tool, with 28 different types identified; 17 of which were specifically designed to assess sleep. Multiple tools within a single CGA were used in 16.4% sources. Bespoke tools were developed in five studies (Table 1).

**Table 1:** Sleep assessment tool evaluation

Sleep assessment tool used	Sources	Sleep specific tool?	Measurement	Bespoke?	Sleep question/topic assessed (if not validated sleep assessment tool)
Pittsburgh Sleep Quality Index (PSQI) [157]	**29** [9, 24, 27–31, 33, 37, 39, 43, 44, 47, 48, 55, 57, 65, 68, 77, 80, 82, 85, 87, 88, 91, 94, 109, 155, 158]	Yes	Sleep quality/disturbance	No	–
Epworth Sleepiness Scale (ESS) [159]	**14** [9, 31, 36, 46, 51, 53, 67, 73, 79, 84, 91, 103, 115, 144]	Yes	Daytime sleepiness	No	–
Insomnia Severity Index (ISI) [160]	**12** [26, 29, 53, 54, 59, 73, 86, 92, 99, 103, 106, 144]	Yes	Insomnia severity	No	–
Athens Insomnia Scale [161]	**6** [75, 98, 100, 104, 111, 112]	Yes	Insomnia severity	No	–
Nottingham Health Profile [162]	[74, 117, 152]	No	Sleep quality/disturbance	No	I take pills to help me sleep. (Y/N) It takes me a long time to get to sleep. (Y/N) I sleep badly at night. (Y/N) I’m tired all the time. (Y/N)
EORTC QLQ-C30 [163]	[132, 154]	No	Sleep quality/disturbance	No	During the past week, have you had trouble sleeping? During the past week, were you tired?
Jenkins Sleep Scale [164]	[31, 113]	Yes	Sleep quality/disturbance	No	–
Berlin Questionnaire [165]	[115]	Yes	Sleep-disordered breathing risk	No	–
Richards- Campbell Sleep Questionnaire [166]	[46]	Yes	Sleep quality/disturbance	No	–
Sleep Hygiene Index [167]	[31]	Yes	Sleep hygiene behaviours	No	–
Cornell Scale for Depression in Dementia [168]	[83]	No	Sleep quality/disturbance	No	Difficulty falling asleep later than usual for this individual? Multiple awakenings during sleep? Early-morning awakening earlier than usual for this individual?
VALINTAN (online CGA software) [169]	[149]	No	Not specified.	Yes	Not specified.
Ten-point visual analogue scale (VAS) for sleep quality evaluation [102]	[102]	Yes	Sleep quality/disturbance	Yes	Not specified.
NoSAS score [170]	[115]	Yes	Sleep-disordered breathing risk	No	–
Patient Health Questionnaire (PHQ-9) [171]	[172]	No	Sleep quality/disturbance	No	Over the last 2 weeks, how often have you been bothered by: trouble falling or staying asleep, or sleeping too much?
Insomnia symptom scale [137]	[137]	Yes	Insomnia severity	Yes	Not specified.
STOP- Bang questionnaire [173]	[38]	Yes	Sleep-disordered breathing risk	No	–
Sleep disturbance scale [174]	[25]	Yes	Sleep quality/disturbance	No	Difficulty falling asleep, taking or being dependent on medication to help one sleep, sleep interrupted during the night, difficulty sleeping (falling/staying asleep) owing to moods or tension, difficulty sleeping owing to pain or itching, inability to return to sleep after waking at night, waking early or feeling tired and sleeping more than 2 hours during the day.
REM Behaviour Disorder Single-Question Screen (RBD1Q) [175]	[25]	Yes	Primary sleep disorders	No	–
InterRAI Long-Term Care Facilities [176]	[90]	No	Sleep quality/disturbance	No	Difficulty falling asleep or staying asleep, waking up too early, restlessness, non-restful sleep, too much sleep, time asleep during the day.
Standardised Assessment for Elderly Patients in Primary Care (STEP) [177]	[72]	No	Not specified.	No	Not specified.
Sleep Habits Questionnaire [42]	[42]	Yes	Sleep quality/disturbance, Primary sleep disorders, sleep-disordered breathing risk	Yes	Questions about perceived sleep (difficulty sleeping, waking up during the night, difficulty to get back to sleep and waking up too early in the morning). Daytime characteristics described by the patients were tiredness, sleepiness and lack of enough sleep. The participants included also asking about some unusual behaviour during sleep, such as leg movements, excessive snoring and pauses in breathing.
MD Anderson Symptom Inventory (MDASI) [178]	[124]	No	Sleep quality/disturbance	No	How severe is your disturbed sleep at is worst? How severe is your feeling drowsy at is worst?
ICSD: International Classification of Sleep Disorder [179]	[141]	Yes	Primary sleep disorders	No	–
Self-reported questionnaire assessed sleep characteristics [95]	[95]	Yes	Sleep quality/disturbance, primary sleep disorders, sleep-disordered breathing risk	Yes	Average total sleep time; time of getting up; time of going to bed. Feeling of poor-quality sleep; use of sleep medication; nocturnal walking for urination; trouble falling asleep after nocturnal waking, waking too early, waking not rested. Restless legs; leg cramps; increased dream; sleep paralysis; sleep talking; teeth-grinding; snoring.
Eysenck’s Personality Inventory (EPI) [180]	[48]	No	Sleep quality/disturbance	No	Do ideas run through your head so that you cannot sleep? Do you suffer from sleeplessness?
Centre for Epidemiologic Publications Depression Scale-8 (CES-D-8) [181]	[48]	No	Sleep quality/disturbance	No	During the past week, my sleep was restless (0–3). Often having trouble falling or staying asleep?
The Beck Depression Inventory (BDI) [182]	[102]	No	Sleep quality/disturbance	No	I can sleep as well as usual. (0) I don’t sleep as well as I used to. (1) I wake up 1–2 hours earlier than usual and find it hard to get back to sleep. (2) I wake up several hours earlier than I used to and cannot get back to sleep. (3)

The Pittsburgh Sleep Quality Index (PSQI), which measures an individual’s sleep quality over the previous month [157], was the most commonly used assessment tool (21.3%). The Epworth Sleepiness Scale (ESS) was the second most used tool (10.3%), often used in practice to assess the severity of daytime symptoms in those with suspected obstructive sleep apnoea syndrome [183]. Insomnia Severity Index (ISI), which asks users to rate the severity of symptoms using a Likert-type scale [160], was the third most frequently used tool (8.8%). All are widely translated into different languages [184–186], supporting international use. These tools were predominantly used in primary research (34.5%) compared to grey literature (2.9%).

General questions about sleep were used in 42.6% of sources. In 81.6% of these, such questions replaced the use of a validated tool, with the remainder combining both approaches. Questions addressed various domains, including sleep quality, sleep duration, insomnia complaints (difficulties initiating/maintaining sleep, early awakenings), daytime sleepiness, restless legs, night-time awakenings, snoring, nocturia, sleep medication use and sleep hygiene. The most frequent question concerned difficulty falling asleep: ‘Do you usually have trouble falling asleep?’ [32], ‘Does it take more than 30 minutes to fall asleep?’ [93], ‘Do you have trouble falling asleep…?’ [89], ‘How long until you fall asleep?’ [187] and ‘Any history of…difficulty falling asleep…?’ [188].

A small proportion (3.7%) [156, 189–192] did not use a tool or questions but mentioned sleep within the text.

### Practitioner involvement and care setting

Where stated, nurses and geriatricians were most involved in assessing sleep within the CGA (Table 2). Care settings were defined as either primary, secondary, tertiary or community [193]. Primary care included general practice and community health centres, while secondary care included hospital inpatients, outpatients and the emergency department. Tertiary care settings referred to studies conducted in a specialised healthcare centre or if this term was used specifically within the publication. Community settings included the patient’s own home and care home environment. Sleep assessment was mostly performed in secondary care settings (55.9%), followed by the community setting (16.9%), tertiary care (10.3%) and primary care (9.6%). The setting was not specified in 4.4% sources and in 2.9%, a combination of primary and secondary care settings was noted. Within the secondary care setting, outpatient clinics were the most common location for the CGA (30.9%). A total of 61.9% of these were geriatric outpatient clinics.

**Table 2:** Count of healthcare practitioners involved in sleep assessment

Practitioner involved	Count
Not specified	86
Nurse/nurse practitioner	17
Geriatrician	16
Physician/doctor	9
Multidisciplinary team	5
Research staff	5
Dietician/nutritionist	3
Psychologist	3
Therapists (physio/occupational)	5
Pharmacist	2
General practitioner	2
Secondary care specialist doctor	3
Social care specialist	1
Care coordinator	1
Elder life specialist	1
Trained volunteer	1
Psychiatrist	1
Gerontologist	1
Geriatric assessor	1
Case manager	1
Experienced clinician	1
Health and social care worker	1

Turkey was the country most represented by the literature (38 sources), followed by Canada and China equally in 12 sources (Supplementary Appendix 3).

### Participant comorbidities

Comorbidities of participants were recorded in 65.4% sources. The most common were hypertension (42.7%), diabetes (34.8%) and coronary artery disease (41.0%). Patients with cancers were represented in 14.6% studies.

### Reasons for exclusion

Exclusion criteria were noted in 57.4% sources. The most common reasons were cognitive impairment and/or dementia (32.4%), severe/acute/unstable illness (19.1%), hearing/vision impairment (14.0%) and severe mental illness (13.2%).

### Results of individual sources of evidence

The completed data charting table is provided in Supplementary Information.

## Discussion

Insomnia is a common global problem that increases in prevalence with advancing age [194]. This scoping review found that sleep is being assessed as part of the CGA in both research and practice. However, approaches were highly variable in terms of methods used, settings and the practitioners delivering the assessment.

The diversity of tools and questionnaires across the included studies reflect considerable variation in practice approaches, assessment priorities and methodological frameworks. In clinical practice, choice of assessment tool may be guided by the aspect of sleep of most interest, or for determining the risk for a particular mechanism causing excessive sleepiness, such as obstructive sleep apnoea [195]. Importantly, a distinction should be made between sleep assessment conducted as part of routine CGA practice and sleep measures incorporated within research studies that utilise CGA domains. In research contexts, sleep may be included to address specific study aims using validated instruments that are not routinely implemented in practice which could reflect research priorities rather than real-world CGA delivery. This distinction is important when interpreting the extent to which sleep is systematically embedded within routine CGA frameworks and when considering conclusions regarding standardisation and feasibility.

Within primary research studies, the array of sleep assessment tools used is likely to represent individual study design methodology and appropriateness for the study population. For instance, 21 different tools were applied only once, limiting generalisability. Ten tools were not designed for sleep, instead capturing symptoms indirectly, often through depression or cancer-related assessments [196, 197]. The common morbidities of participants in the review are cardiometabolic conditions which have known bidirectional links with disturbed sleep [198] as well as cancer, in which disturbed sleep is caused by both the disease and treatment [199].

In 42.6% sources, sleep was assessed using general, non-validated questions rather than formal tools. In most cases (75.9%), the specific questions were not reported. This demonstrates a nonstandardised approach and warrants the need for clearer guidance on sleep assessment within the CGA.

The PSQI was most commonly used validated tool, extensively measuring multiple domains of sleep [157]. It has already been studied in a range of populations, including older people [200]. However, its suitability in those with cognitive impairment may not be appropriate, given the need for recall of the previous month’s sleep which could render self-report less accurate.

The most common general screening question was based on iterations of ‘Do you have difficulty falling asleep?’. This may be useful as an initial screening item, but risks missing those with sleep maintenance or early awakening difficulties if used in isolation. Future research should explore populations likely to benefit from more detailed assessment, rather than relying on screening questions. Additionally, validation of screening tools alongside objective assessment [201] in populations with cognitive impairment is needed, as this group was most often excluded from reviewed studies.

The role of the sleep assessor was frequently unspecified. When identified, assessors were most commonly nurses, nurse practitioners or geriatricians—key members of the multidisciplinary team [202]. The range of professions involved demonstrates that sleep assessment could be embedded within the skillset of any professional delivering CGA. However, the absence of clearly defined roles may limit consistent implementation. Promoting the importance of sleep assessment within undergraduate and postgraduate training across disciplines would encourage the use of sleep assessments as standard practice [203–205]. Furthermore, frailty teams could designate appropriate practitioners within the multidisciplinary team to undertake sleep assessment, supported by targeted training where required. Clear referral pathways, follow-up processes and access to appropriate support services would also be necessary to ensure that identified sleep problems are appropriately managed.

Sleep was assessed across all healthcare settings, supporting its feasibility. Most assessments were carried out in outpatient clinics with clinically stable patients. In contrast, inpatient assessment may be more challenging due to acute physical or mental illness or the constraints of busy, crowded emergency departments where time to form holistic, patient-centred plans is limited [206]. Primary care face barriers such as limited consultation time and workload [207]. It is acknowledged that completing a CGA is already an extensive process; therefore, shorter adapted tools such as the ISI-3: a three-item version of the standard ISI, validated for use in the older people [208], may offer an efficient and practical alternative in these contexts. Furthermore, the practitioner may choose appropriate screening questions tailored to their assessment, as a range of different examples were included in the review. This could help identify those with sleep problems affecting daytime function, especially if the patient is concerned about the impact of this.

International variation of sleep assessment practice was identified. Turkey most frequently reported sleep assessment in older people, with studies recommending that sleep quality assessment should be included within a CGA [25, 37]. The country also has strong sleep research activity and has a well-established Sleep Medicine Society [209]. It does not, however, operate a single, nationally mandated CGA template that explicitly lists sleep as a core domain. Instead, sleep was usually assessed sleep as a subarea of CGA, much like falls or incontinence. Canada recognises sleep within core CGA frameworks [188, 192], while China classifies sleep disturbance as a geriatric syndrome [60]. In contrast, current UK guidance does not include sleep assessment as either a core domain or subarea of CGA [202]. This disparity illustrates how national guidance and policies shape CGA delivery and contributes to inconsistent practice across healthcare systems. Although no international consensus statement currently mandates inclusion of sleep within CGA, practice patterns suggest increasing recognition of its clinical relevance given the clinical consequences of sleep disturbance in older adults. Development of consensus guidance outlining minimum standards for sleep assessment within CGA may provide a framework to support policymakers and improve standardisation across healthcare systems. Inclusion of sleep assessment as a core domain (or subdomain) of CGA would help ensure the systematic identification of sleep problems in routine practice and facilitate standardised care.

### Limitations

Most primary research studies included in this review excluded certain patient groups, such as those with cognitive impairment, resulting in those at higher risk of sleep problems being underrepresented. Common tools like PSQI, ESS and ISI which have multiple items [157, 159, 160], requiring participant attention, understanding and recall have not been validated in people with dementia and as such may limit applicability to this population [210]. The lack of capacity to report sleep disturbance is a widely recognised issue in practice [211]. Pragmatic approaches such as questioning the patient’s bed partner, carer or next-of-kin about sleep habits and disturbances may support problem recognition. Use of actigraphy may also be explored in this population [212] as a less invasive approach to polysomnography for formal sleep assessment. The latter would not be feasible for routine CGA implementation due to limited resource availability.

The search strategy included both CGA and sleep terms in published sources which would have excluded undocumented real-world practice. Furthermore, the review focused on CGAs that explicitly included sleep assessment alongside established physical, functional, psychological, social and environmental domains. Consequently, the relative frequency of sleep assessment within overall CGA practice could not be determined. However, sleep was less consistently reported than core CGA domains in national surveys [213, 214]. An audit of contemporary CGA practice may help quantify current uptake and identify examples of good practice that could inform wider implementation. Some studies assessed sleep as the primary focus of the research, which may overestimate its inclusion in routine CGA. Finally, the review only explored literature in the English language therefore may underrepresent practice in non-English speaking countries.

Despite these limitations, this review provides a synthesis of available and published sleep-related assessment methods within the CGA. This information is valuable for clinicians planning services, researchers designing applied clinical studies and policy-makers involved in the development of holistic geriatric care frameworks.

## Conclusions

Sleep assessment within CGA varies globally. The most frequently used tool was the PSQI. Assessment questions regarding sleep problems could prompt a more holistic assessment of health and wellbeing in the older person, which could feasibly be performed within a CGA by any practitioner. Clearer guidance defining the standards for routinely incorporating sleep into CGA and training across healthcare professions would promote implementation, ensuring that sleep problems are recognised and addressed as part of holistic geriatric care.

## Supplementary Material

aa-25-3368-File002_afag094

## Data Availability

Research data is available in Supplementary Information.
