# Supplementary material for: Assessment of sleep in older people within comprehensive geriatric assessments: a scoping review
Source: Age Ageing. 2026 May 26;55(5):afag094. doi: 10.1093/ageing/afag094 (PMC13201258; doi:10.1093/ageing/afag094)
Supplement: aa-25-3368-File002_afag094 [file aa-25-3368-file002_afag094.docx]

# Assessment of Sleep in Older People within Comprehensive Geriatric Assessments: A Scoping Review

## Supplementary Data File

## Contents Table

| Content |
| --- |
| Appendix 1: Search strategies |
| Appendix 2: Data charting table |
| Appendix 3: Graph to show countries in scoping review |
| Appendix 4: Excluded publications |
| Appendix 5: PRISMA-ScR Checklist |

## Appendix 1: Search strategies

### Table 1: Search strategy for Medline and PsychInfo (EBSCO)

| (MH "Geriatric Assessment") |
| --- |
| XB comprehensive N3 "geriatric assessment" |
| 1 OR 2 |
| (MH "Aged") |
| (MH "Frail Elderly") |
| (MH "Geriatrics") |
| (MH "Aged, 80 and over") |
| XB "older person" |
| XB elder* |
| XB geriatric* |
| XB "older people" |
| XB "older adult*" |
| XB senior* |
| XB "frail elderly" |
| 3 or 4 or 5 or 6 or 7 or 8 or 9 or 10 or 11 or 12 or 13 |
| XB sleep N3 assessment |
| XB sleep* |
| (MH "Sleep") |
| (MH "Sleep Initiation and Maintenance Disorders") |
| XB insomnia* |
| XB sleep N3 disorder* |
| XB "sleep disturbance*" |
| XB sleepless* |
| XB parasomnia* |
| XB "sleep apnea" |
| XB "sleep apnoea" |
| XB "dyssomnia*" |
| (MH "Insomnia, Fatal Familial") |
| 15 or 16 or 17 or 18 or 19 or 20 or 21 or 22 or 23 or 24 or 25 or 26 or 27 |
| 3 AND 14 AND 28 |

### Table 2: Search strategy for CINAHL and Embase (Ovid)

| Geriatric Assessment / |
| --- |
| comprehensive ADJ3 "geriatric assessment".tw. |
| XB "geriatric evaluation" |
| XB "geriatric consultation" |
| Frail Elderly/ |
| Geriatrics/ |
| Very Elderly / |
| "older person".tw. |
| elder*.tw. |
| geriatric*.tw. |
| "older people".tw. |
| "older adult*".tw. |
| senior*.tw. |
| "frail elderly".tw. |
| 3 or 4 or 5 or 6 or 7 or 8 or 9 or 10 or 11 or 12 or 13 or 14 |
| sleep ADJ3 assessment.tw. |
| sleep* .tw. |
| Sleep/ |
| sleep disorder/ |
| insomnia*.tw. |
| sleep ADJ3 disorder*.tw. |
| "sleep disturbance*".tw. |
| sleepless*.tw. |
| parasomnia*.tw. |
| "sleep apnea".tw. |
| "sleep apnoea".tw. |
| dyssomnia*.tw. |
| insomnia/ or fatal familial insomnia/ |
| sleep terms combined with OR |
| 4 AND 15 AND 29 |

### Table 3: Search strategy for Web of Science

| "geriatric assessment" OR comprehensive NEAR/3 "geriatric assessment" OR  "geriatric consultation" OR "geriatric evaluation" |
| --- |
| Aged OR "Frail Elderly" OR "older person" OR elder*OR geriatric* OR "older people" OR  "older adult*" OR senior* |
| sleep NEAR/3 assessment OR sleep OR "sleep disorder" OR insomnia* OR sleep NEAR/3 disorder*  OR "sleep disturbance*" OR sleepless* OR parasomnia* OR "sleep apnea" OR "sleep apnoea" OR dyssomnia* |

## Appendix 2: Data charting table

| **Year** | **Title** | **Country** | **Setting** | **Identification: Comments** | **Author's name** | **Methods: Aim/Purpose of study or guideline** | **Methods: Type of evidence source** | **Methods: Was sleep assessment main focus of study/guideline? (Yes/No)** | **Population: Exclusion criteria** | **Sample size (people)** | **Age range (years)** | **Mean age ± SD (years)** | **Median age (min-max) years** | **Co-morbidities (list up to 3 from study if reported- highest frequency to lowest)** | **Assessment tool or questionnaire to assess sleep** | **Were general questions asked about sleep? (in addition to tool or instead of using tool)** | **Which questions were asked about sleep? List here or type "not stated".** | **If patient was asked about their sleep but questions aren't listed, record what patient was asked about.** | **Practitioner(s) involved in assessment.** |
| --- | --- | --- | --- | --- | --- | --- | --- | --- | --- | --- | --- | --- | --- | --- | --- | --- | --- | --- | --- |
| 1997 | Sleep disturbance, psychosocial correlates, and cardiovascular disease in 5201 older adults: the Cardiovascular Health Study... abstract presented at the November 1993 meeting of American Geriatrics Society. | USA | Community | Sample of the Health Care Finance Administration Medicare eligibility lists in four US communities | Newman et al. | To describe the prevalence of self reported sleep disturbance in older men and women and report the association of these symptoms with other markers of physical and psychologic health. | Primary research | Yes | Institutionalised, wheelchair bound in the home, or currently under treatment for cancer. | 5201 | 65-100 | 72.8 ± 5.6 | - | Heart disease and stroke | - | Yes | Three questions were included to assess symptoms of difficulty initiating and maintaining sleep, including difficulty falling asleep, frequent awakenings, and waking up too early, modified from a questionnaire assessing sleep quality.” Daytime sleepiness was also assessed. The possible responses were limited to “yes, ” “no, ” or “don’t know” (categorical variables coded as 1, 0, or missing, respectively). Q1. Are you usually sleepy in the daytime? (Daytime Sleepiness) Q2. Do you usually have trouble falling asleep? (Difficulty Falling Asleep) Q3. Do you usually wake up several times at night? (Frequent Awakenings) Q4. Do you usually wake up far too early? (Wake Up Too Early) | - | Not specified |
| 1998 | The association between chronic illness and functional change among participants in a comprehensive geriatric assessment program | USA | Community | - | Cho et al. | To examine the association between chronic illness and functional status change during a 3-year period in older people enrolled in an in-home comprehensive geriatric assessment (CGA) and preventive care program. | Primary research | No | severe cognitive impairment, language problems, plans to move to a nursing home, plans to move away, self-reported terminal disease, participation in another randomized trial, or severe functional impairment | 202 | ≥75 | 80.8 ± 3.8 | - | - | - | Yes | Not stated | Sleep disorder | Gerontologic nurse practitioners and geriatricians |
| 2000 | The Hospital Elder Life Program: A model of care to prevent cognitive and functional decline in older hospitalized patients | Canada | Secondary care | Hospital Elder Life Program | Inouye et al. | To describe the Hospital Elder Life Program, a new model of care designed to prevent functional and cognitive decline of older persons during hospitalization. | Primary research | No | - | 1507 | 70-100 | 81 | - | - | - | Yes | Not stated | Quality of sleep; the use of sedative drugs | Geriatric nurse specialist, Elder Life Specialists, trained volunteers, and geriatricians-who work closely with primary nurses |
| 2001 | National Service Framework for Older People | UK | Primary and secondary care | All care settings for older people | Department of Health | To support independence and promote good health, specialised services for key conditions, and culture change so that all older people and their carers are always treated with respect, dignity and fairness. | National service framework for older people | No | - | - | ≥ 65 | - | - | - | - | Yes | Not stated | Sleeping patterns (self assessment) | Not specified |
| 2007 | Postoperative delirium in old patients with femoral neck fracture: A randomized intervention study | Sweden | Secondary care | Postoperative care in a specialized geriatric ward or a conventional orthopedic ward | Lundström et al. | To investigate whether a postoperative multi-factorial intervention program, including comprehensive geriatric assessment, management and rehabilitation, can reduce delirium and improve outcome in patients with femoral neck fractures. | Primary research | No | age under 70, severe rheumatoid arthritis, severe hip osteoarthritis, severe renal failure, pathological fracture, and patients who were bedridden before the fracture | 199 | ≥ 70 | 82 ± 6 | - | Cardiovascular disease, hypertension, osteoporosis | - | Yes | Not stated | Sleeping problems | Nurses, an occupational therapist, a physiotherapist and physicians. |
| 2009 | Developing a comprehensive interdisciplinary Healthcare Program for the elderly in senior residence | South Korea | Community | Senior residence | Lee et al. | To consider the epidemiology of the diseases (malignant neoplasm, cerebrovascular disease, heart disease and diabetes mellitus), needs of the elderly and evidence-based approaches for the construction of health screening items. | Conference abstract | No | - | 33 | ≥ 65 | 67.4 | - | Coronary artery stenosis, ischemic changes in the cerebral white matter and cerebrovascular occlusions; osteoporosis. | ICSD: International Classification of Sleep Disorder | No | - | - | Multidisciplinary team |
| 2011 | Cross sectional geriatric assessment of Mexican older people | Mexico | Secondary care | Outpatients of Family Medicine Units | Cortés et al. | To report the results of an integral geriatric evaluation of Mexican older people. | Primary research | No | - | 324 | ≥ 60 | - | 70 (59-71) | - | - | Yes | Does it take more than 30 minutes to fall asleep? At night, do you sleep less than 6 hours? When you wake up in the morning, do you feel tired? | - | Not specified |
| 2011 | Use of comprehensive geriatric assessment in general practice: Results from the 'Senta Pua' project in Brazil | Brazil | Secondary care | Hospital outpatients | Lucchetti and Granero | To evaluate (a) whether a CGA can identify previously unknown health problems, and (b) whether a CGA is accepted by elderly patients. | Primary research | No | Patients with cognitive impairment precluding completion of the test (advanced dementia or inability to communicate) or aged younger than 60 years were excluded. | 170 | ≥ 60 | 75.7 ± 8 | - | - | - | Yes | Do you suffer from frequent sleeping problems? | - | General practitioners |
| 2012 | Screening postacute care needs of hospital inpatients in Taiwan: A hospital-based study | Taiwan | Tertiary care | Post acute care | Chou et al. | To evaluate the postacute care (PAC) needs of elderly patients in a tertiary teaching hospital. | Primary research | No | Patients admitted for elective procedures, acute conditions related to terminal illness, including malignancy, and considered to have a low potential for functional recovery, were excluded. | 409 | >65 | 80.4 ± 5.4 | - | Musculo- skeletal conditions, infectious diseases and central nervous system conditions. | - | Yes | Not stated | Sleep problems | Trained case managers and geriatricians |
| 2012 | Psychological distress as a key component of psychosocial functioning in community-dwelling older people | Ireland | Community | Geriatric assessment clinic | Schnittger et al. | To explore the key components representative of measures of psychosocial functioning with a focus on identifying the constituents of psychological distress in an Irish sample of community-dwelling older adults and to examine the relationship between these components and health outcomes such as frailty. | Primary research | No | - | 579 | 60-92 | 72.5 ± 7.1 | - | - | Pittsburgh Sleep Quality Index, Eysenck’s Personality Inventory (EPI), Center for Epidemiologic Studies Depression Scale-8 (CES-D-8) | Yes | Do you suffer from sleeplessness? (from EPI) Much of the time during the past week, your sleep was restless (from CES-D-8). | - | Physicians and psychologists |
| 2013 | Does self-reported sleep quality predict poor cognitive performance among elderly living in elderly homes? | Egypt | Community | Elderly home | Amer et al. | To study the relation between sleep quality and cognitive performance in older adults living in elderly homes. | Primary research | Yes | Cerebrovascular disease, known depression or scoring 5 or more in the GDS and taking any psychotropic or sedative hypnotic medications, also who refused or who were not cooperative. | 100 | ≥ 60 | 65 ± 3.4 | - | Hypertension, Diabetes Mellitus, Chronic liver disease | Pittsburgh Sleep Quality Index | No | - | - | Experienced Clinician |
| 2014 | Development and usefulness of a new questionnaire for assessing geriatric syndrome | Japan | Secondary care | Outpatient memory clinic | Nagi et al. | To assess the validity and reliability of a pre-visit questionnaire newly developed to identify geriatric conditions in older adults in an outpatient clinical setting. | Primary research | No | - | 277 | - | - | - | - | - | Yes | "Do you have trouble falling asleep, wake up during the night, or take sleeping pills?" Y/N | - | Not specified |
| 2014 | Factors associated with increased caregivers' burden in several cognitive stages of Alzheimer's disease. | Japan | Tertiary care | National Center for Geriatrics and Gerontology | Kamiya et al. | To identify the factors associated with Care Burden according to the stage of cognitive decline in older adults with Alzheimer's disease (AD) | Primary research | No | Patients with severe conditions, such as cardiac failure, renal disorder, liver dysfunction, neurological and psychiatric disorders such as depression, and alcohol abuse. | 1127 | - | 78.5 ± 6.2 | - | Alzheimer's disease | - | Yes | Not stated | Daytime sleepiness and sleep disturbance | Not specified |
| 2014 | Functional assessment of geriatric patients in regard to health-related quality of life (HRQoL) | Poland | Secondary care | Geriatric Clinic inpatients | Muszalik et al. | To evaluate general health, functioning, and performance parameters, as well as care problems of Geriatric Clinic inpatients in relation to deficits in fulfilling needs. | Primary research | No | - | 227 | ≥ 60 | 73.6 | - | Cardiac diseases, metabolic diseases, respiratory system diseases | Nottingham Health Profile | No | - | NHP questionnaire consists of 5 questions on sleep. sleep disturbances in the form of difficulties in falling asleep and the need to use sleeping pills. | Not specified |
| 2014 | Palliative care needs: Symptom reporting during geriatric oncology evaluation | USA | Community | Geriatric oncology consult clinic | Flannery et al. | To identify geriatric oncology patients' symptom reports, the number of symptoms experienced, and interference reported from symptoms. | Conference abstract | No | - | 192 | 65-95 | - | 81 | - | MD Anderson Symptom Inventory (MDASI) | No | - | - | Not specified |
| 2014 | Comprehensive geriatric assessment reveals sleep disturbances in community-dwelling elderly adults associated with even slight cognitive decline | Japan | Community | Rural Japanese town | Otsuka et al. | To investigate whether there were significant associations between mild cognitive decline and poor sleep quality indicators in older citizens in a rural Japanese town. | Primary research | Yes | Age <75 years, Mini-mental state examination (MMSE) <25 | 691 | 75-102 | 81 ± 4.6 | - | Cognitive impairment, hypertension, dizziness | A self-reported questionnaire assessed sleep characteristics | Yes | Not stated | Average total sleep time; time of getting up; time of going to bed. Feeling of poor-quality sleep; use of sleep medication; nocturnal walking for urination; trouble falling asleep after nocturnal waking, waking too early, waking not rested. Restless legs; leg cramps; increased dream; sleep paralysis; sleep talking; teeth- grinding; snoring. | Not specified |
| 2015 | Frailty screening in the community using the FRAIL scale | Hong Kong | Community | Elderly Centres | Woo et al. | To explore the feasibility of using the FRAIL scale in community screening of older Chinese people aged 65 years and older, followed by clinical validation by comprehensive geriatric assessment of those classified as pre-frail or frail. | Primary research | No | - | 816 | ≥ 65 | 76.3 ± 7.4 | - | - | - | Yes | Not stated | Night-time sleep duration, self-reportedinsomnia, the use of sleeping pills | Multidisciplinary team |
| 2015 | Risk factors for depression among elderly subjects with hypertension living at home in China | China | Community | Patient's home | Ma et al. | To determine the risk factors for depression in a hypertensive population residing in Beijing in 2004. | Primary research | No | Secondary hypertension, dementia, or inability to answer the questions. | 1064 | ≥ 60 | - | - | Hypertension | - | Yes | sleep quality (well/not well) | - | Not specified |
| 2015 | Malnutrition is associated with dementia severity and geriatric syndromes in patients with Alzheimer disease. | Turkey | Secondary care | Outpatient neurology clinics | Yildiz et al. | To screen community-dwelling Alzheimer disease patients for malnutrition and geriatric syndromes and to determine clinical factors associated with malnutrition in these patients. | Primary research | No | Significant kidney dysfunction (a creatinine level of >1.5 mg/dL), comorbidities other than dementia (like malignancy) that may be associated with malnutrition, and an established diagnosis of malnutrition under treatment. | 76 | ≥ 65 | 79 ± 7.4 | - | Alzheimer disease | - | Yes | Not stated | Normal/abnormal sleep | Not specified |
| 2015 | Relationship between perceived sleep and polysomnography in older adult patients | Brazil | Community | Neighborhood of Vila Clementino, district of Sao Paulo. | dos Santos Silva et al. | To compare perceived sleep with the polysomnography of older adult evaluated at the Center for the Study of Aging UNIFESP, given the subjective and objective assessments of sleep may be discrepant due to sleep misperception and measurement effects. | Primary research | Yes | Refusal to perform polysomnography and/or problems that interfere with the examination, such as flu or fever. | 40 | 64-89 | 73.7 | - | - | Sleep Habits Questionnaire | Yes | Not stated | Questions about perceived sleep (difficulty sleeping, waking up during the night, difficulty to get back to sleep and waking up too early in the morning). Daytime characteristics described by the patients were tiredness, sleepiness and lack of enough sleep. The participants included also asking about some unusual behavior during sleep, such as leg movements, excessive snoring and pauses in breathing. | Physician, nutritionist, physical educator and psychologist. |
| 2015 | Association between geriatric assessment tools and self-percieved health-related quality of life measured by Nottingham healh profile | Spain | Secondary care | Geriatric hospital | Argilaga et al. | To assess whether there is an association between the scores obtained from the NHP and the results of the comprehensive geriatric assessment. | Conference abstract | No | - | 331 | - | 77.5 ± 10 | - | - | Nottingham Health Profile | No | - | - | Not specified |
| 2016 | Comprehensive Geriatric Assessment Template | Canada | Community | CGA Toolkit | Peel | These tools will help healthcare providers pull together a comprehensive clinical picture, and ensure all of the common geriatric syndromes are being addressed. | Assessment template | No | - | - | - | - | - | - | - | Yes | Not stated | Sleep: ex. snoring/apnea/unrested, daytime napping, night time routine, difficulty falling asleep vs wakenings, use of sleep aids | Not specified |
| 2016 | Comprehensive Geriatric Assessment Form | Canada | - | Assessment form | Rockwood | Comprehensive geriatric assessment assessment form for practice | Comprehensive geriatric assessment form | No | - | - | - | - | - | - | - | Yes | Not stated | Sleep: normal, disrupted, daytime drowsiness | Not specified |
| 2016 | A geriatric assessment in general practice: prevalence, location, impact and doctor-patient perceptions of pain | Germany | Primary care | General practice | Kruschinski et al. | To investigate what a geriatric assessment in general practice adds towards previous findings of prevalence, location, impact and the dyadic doctor-patient perception of pain in this age group. | Primary research | No | Long-term care dependency level II or III, dementia, limited contractual capability or incapacity, insufficient language skills, severe hearing loss, current participation in another clinical trial, and no availability by telephone | 297 | ≥ 70 | 77 ± 5 | - | Pain | Standardised Assessment for Elderly Patients in Primary Care (STEP) | Yes | Not stated | Sleeping disorder | Study nurse |
| 2016 | Interprofessional Comprehensive Geriatric Assessment Toolkit | Canada | - | CGA Website | Peel | To provide some foundational information and learning resources related to Comprehensive Geriatric Assessment. | Toolkit | No | - | - | - | - | - | - | - | Yes | Do you have any problems with sleep? If you answered yes to the above:  Any history of snoring/nocturnal choking and gasping or unrestful sleep? Any history of daytime napping, difficulty falling asleep, or multiple awakening’s during sleep, use of sleep aids or over the counters for sleep? How many times do you wake to urinate at night?  Do you have any unusual behaviours or movements during sleep? Are your legs restless while you are trying to fall asleep? What do you do before going to sleep (caffeine, stimulating activities)? | - | Not specified |
| 2017 | A Competency Framework for Interprofessional Comprehensive Geriatric Assessment | Canada | Primary and secondary care | Competency Framework for use across settings | Regional Geriatric Programs of Ontario | The purpose of this competency framework is to describe detailed practice expectations of health professionals participating in the CGA. | Competency framework | No | - | - | - | - | - | - | - | Yes | Not stated | Changes in sleep patterns. Sleep Apnea | Geriatric assessor |
| 2017 | A prospective study to evaluate the utility of geriatric assessment and intervention in patients with lymphoproliferative disorders in a tertiary hospital | Spain | Tertiary care | Tertiary hospital | Bastos‐Oreiro et al. | To evaluate the utility of geriatric assessment in older patients with lymphoproliferative disorders. | Conference abstract | No | Patients considered not eligible for treatment | 36 | ≥ 70 | - | 77 (75-81) | Lymphoproliferative disorders | - | Yes | Not stated | Sleep disturbance | Not specified |
| 2017 | Comprehensive geriatric assessment in elderly patients with severe aortic stenosis referred for transcatheter aortic valve implantation (TAVI) | Spain | Secondary care | - | Roqueta et al. | To assess patients with severe symptomatic aortic stenosis with a comprehensive geriatric assessment. | Conference abstract | No | - | 45 | - | 82.6 ± 6 | - | Severe aortic stenosis | - | Yes | Not stated | Insomnia | Geriatrician |
| 2017 | Health-related quality of life in elderly patients with haematological malignancies - High prevalence of fatigue and pronounced impact on survival | Austria | Secondary care | Department of Internal Medicine | Hofer et al. | To analyse of impact of blood cancer on assessment status and on QoL in advanced age on assessment status and clinical outcome. | Conference abstract | No | - | 149 | ≥ 70 | - | - | Haematological malignancies | EORTC QLQ-C30 | No | - | - | Not specified |
| 2017 | Determinants of late-life depression in residents of long-term care facility | Poland | Community | Longterm care facilities (LTCF) | Horwath and Szczerbińska | To assess the prevalence and identify factors related to late-life depression symptoms in long-term care facilities (LTCF) residents, with a goal of improving recognition and expanding the awareness of late-life depression in LTCF staff. | Primary research | No | Those in whom it was not possible to complete the questionnaire due to the lack of a discernible consciousness or the presence of coma or in account of lack of data on analysed factors. | 290 | 31-94 | - | 79 (67-86) | Depression, Congestive heart failure, Psychiatric disorders other than depression | InterRAI Long-Term Care Facilities | No | - | - | Nurses |
| 2017 | Aging in HIV-infected and uninfected populations: A comprehensive geriatric assessment | Spain | Primary and secondary care | Primary Care Centre and HIV unit | Negredo | To evaluate a comprehensive geriatric assessmennts in HIV-infected and uninfected populations. | Conference abstract | No | - | 91 | - | - | - | HIV | Pittsburgh Sleep Quality Index | No | - | - | Not specified |
| 2017 | An under-diagnosed geriatric syndrome: Sleep disorders among older adults | Turkey | Secondary care | Geriatrics outpatient clinic | Tufan et al. | To determine the prevalence of sleep problems among older adults admitted to the geriatrics outpatient clinic. | Primary research | Yes | Patients who received <24 for the Mini-Mental State Examination (MMSE) and/or did not agree to participate. | 203 | ≥ 75 | 80.9 ± 4.3 | - | Hypertension, Osteoporosis, Hyperlipidemia | Sleep disturbance scale, REM Behaviour Disorder Single-Question Screen (RBD1Q) | Yes | "Do you feel discomfort during some nights that causes an urge to move your legs?" | Difficulty falling asleep, taking or being dependent on medication to help one sleep, sleep interrupted during the night, difficulty sleeping (falling/staying asleep) owing to moods or tension, difficulty sleeping owing to pain or itching, inability to return to sleep after waking at night, waking early or feeling tired, sleeping more than two hours during the day. | Geriatrician |
| 2017 | Associations of sleep disturbance with physical function and cognition in older adults with cancer | USA | Tertiary care | Specialized Oncology Care & Research in the Elderly (SOCARE) clinics | Loh et al. | To examine the prevalence of sleep disturbance and its association with physical function and cognition in older adults with cancer. | Primary research | Yes | - | 389 | 55-97 | 81 ± 6.9 | 81 | Cancer, depression | - | Yes | "Do you have sleep problems now?" (Yes/No) | - | Not specified |
| 2017 | Impact of insomnia on self-perceived health in the elderly. | Brazil | Secondary care | Geriatric outpatient clinic | Silva et al. | To investigate the association between self-perceived health, and sociodemographic and clinical factors in a sample of elderly outpatients in Rio de Janeiro. | Primary research | No | Diagnosis of dementia and who did not answer the question regarding Self-perceived health | 326 | ≥ 60 | - | - | Hypertension, diabetes, oseteoarthritis | - | Yes | Not stated | Insomnia defined against ICD10 criteria. Sleep disorder (yes/no) | Not specified |
| 2017 | Home Based Primary Care for Frail Elderly People | Canada | Community | Home Based Primary Care | Rosenberg | To benchmark practice, follow people longitudinally to study and evaluate interventions. | Research presentation | No | Not living in retirement home, MMSE<20 and not able to consent. | 57 | ≥75 | - | - | - | Pittsburgh Sleep Quality Index | No | - | - | Nurse and research assistant |
| 2018 | Comprehensive Geriatric Assessment: Interprofessional Team Recommendations for Older Adult Women With Breast Cancer | USA | Tertiary care | Geriatric oncology ambulatory care clinic | Overcash | To identify limitations reflected by mean scores on the CGA instruments and describe the CGA recommendations documented in the medical record. | Primary research | No | - |  | 69-93 | 79.1 | - | Breast cancer | Pittsburgh Sleep Quality Index | No | - | - | Geriatric nurse practitioners |
| 2018 | Relationship between sleep duration and coronary heart disease in older adults | Turkey | Secondary care | Geriatric outpatient clinic | Ãœnsal et al. | To examine the relationship between sleep duration and coronary artery disease (CAD) and cardiovascular risk factors. | Primary research | Yes | Patients with advanced dementia, delirium, or other psychiatric disorders who were incomplete in their clinical information, whose sleep durations were not assessed, and who were unable to cooperate with comprehensive geriatric assessment tests. | 2255 | - | 72 ± 6 | - | Hypertension, coronary artery disease, depression | - | Yes | "How many total hours of sleep do you usually get per day?" | Insomnia, difficulty falling asleep and sleep duration. | Not specified |
| 2018 | Evaluation of a standardized geriatric assessment at diagnosis in a prospective cohort of elderly patients with newly diagnosed acute myeloid leukemia | France | Secondary care | - | Saillard et al. | To evaluate the impact of a standardized geriatric assessment at diagnosis in a prospective cohort of newly diagnosed AML in elderly patients, and to investigate correlations between geriatric scores and overall survival. | Conference poster | No | - | 94 | 70-96 | - | 75.5 (70-96) | Acute myeloid leukemia (AML) | Insomnia symptom scale | Yes | Not stated | Insomnia | Not specified |
| 2019 | Rapid Geriatric Assessment, Physical Activity, and Sleep Quality in Adults Aged more than 65 Years: A Preliminary Study | Turkey | Secondary care | Outpatient physical medicine and rehabilitation clinic | Tuna et al. | (1) To evaluate geriatric syndromes using the Rapid Geriatric Assessment; (2) To investigate possible association of geriatric syndomes with physical activity and sleep quality in adults aged more than 65 years who applied to outpatients physical medicine and rehabilitation clinic. | Primary research | Yes | Those with serious communication problems and serious neurological (e.g. abnormal pattern of posture or movement and history of stroke), orthopaedic (e.g., injury and surgery history), rheumatic and vascular disorders that affect walking. | 56 | ≥ 65 | 69.7 ± 4 | 69 (65-80) | - | Pittsburgh Sleep Quality Index | No | - | - | Not specified |
| 2019 | e-GAB: Electronic Geriatric Assessment Bundle: Development of an Arabic Self-administered Android CGA ApplicationA1 - Anonymous. | Egypt | Community | Community-dwelling | Rasheedy | To design and assess the accuracy of administering the electronic Geriatric Assessment Bundle (e-GAB) compared to geriatrician performed comprehensive geriatric assessment (CGA) in a group of Egyptian elderly. | Clinical trial protocol | No | Any patient refused to participate. | 50 | 60-90 | - | - | - | - | Yes | Not stated | Sleep disturbances | Geriatrician |
| 2019 | Sleep quality and its association with frailty indicators in community dwellers older adults | Spain | Primary care | Primary care centre | Burbano et al. | To assess the association between sleep disturbance with clinical and frailty variables in older community-dwellers. | Conference abstract | Yes | - | 164 | - | 81.7 ± 5.6 | - | - | Jenkins Sleep Scale | Yes | Not stated | Self-reported sleep quality (bad/good) | Geriatrician |
| 2019 | Comprehensive Geriatric Assessment | Canada | - | Undergraduate assessment form | University of Toronto | Comprehensive geriatric assessment assessment form for practice | Comprehensive geriatric assessment form | No | - | - | - | - | - | - | - | Yes | “How is your sleep?” “At what time do you usually go to bed?" "How long until you fall asleep?" "Do you wake up in the middle of the night?" "Do you fall back asleep quickly?" "At what time do you wake up?" | - | Not specified |
| 2019 | Prevalance of geriatric syndromes among outpatient clinics patients over 60 years | Turkey | Secondary care | Geriatric outpatient clinic | Aydin | To show the frequency of geriatric syndromes such as malnutrition, falls, sleep problems, urinary incontinence, constipation, frailty, polypharmacy in patients over the age of 60 who applied to our outpatient clinic. | Conference abstract | No | - | 619 | ≥ 60 | - | - | - | - | Yes | Not stated | Presence of sleep problem | Not specified |
| 2019 | Diabetes and common geriatric syndromes | Turkey | Secondary care | Geriatrics outpatient clinic | Kucukdagli | To assess the relationship between diabetes and common geriatric syndromes. | Conference abstract | No | - | 621 | >60 | 78.5 ± 5.7 | - | Diabetes | - | Yes | Not stated | Sleep disorders | Not specified |
| 2019 | Frailty syndrome is associated with changes in peripheral inflammatory markers in prostate cancer patients undergoing androgen deprivation therapy | Spain | Tertiary care | Inpatients of an oncological centre | Navarro-Martínez et al. | To evaluate the role of peripheral inflammation (leukocyte differential count, the proinflammatory cytokines IL-beta, TNF-a, IL-6, IL-8, and the inflammatory markers fibrinogen and C-reactive protein [CRP]) in frailty syndrome in patients with prostate cancer (CaP) undergoing antiandrogen therapy (ADT). | Primary research | No | Severe cognitive impairment (Mini-Mental State Examination [MMSE] score < 21), severe psychiatric disorders or blindness, and acute infections. | 46 | 51-92 | 72.2 ± 9.4 | - | Prostate cancer | Athens Insomnia Scale | No | - | - | Not specified |
| 2019 | Frailty and chronic pain | Turkey | Secondary care | Geriatrics outpatient clinic | Kucukdagli et al. | To assess prevalence of frailty in elder people and it is relationship with other conditions. | Conference abstract | No | - | 1107 | ≥ 60 | 78.5 ± 5.7 | - | Chronic pain | - | Yes | Not stated | Sleep disorders | Not specified |
| 2019 | Implantation of a HELP program in the surgical division of a large tertiary hospital in Israel | Israel | Tertiary care | Large tertiary hospital | Tellem et al. | To present the implementation of the HELP (Hospital Elder Life Program) program in the surgical division of a large tertiary hospital in Tel Aviv, Israel. | Conference abstract | No | Intubated patients, patients with contact/neutropenic or air insulation, unconsciousness and aphasia. | - | - | - | - | - | - | Yes | Not stated | Sleep | Not specified |
| 2019 | Quality of life and geriatric syndromes in elderly people in the republic of Moldova | Moldova | Secondary care | Geriatric inpatients | Deseatnicova et al. | Establishing the frequency and types of geriatric syndromes in elderly and assessing their quality of life | Conference abstract | No | - | 1158 | ≥ 65 | 71.6 ± 0.2 | - | - | Nottingham Health Profile | No | - | - | Not specified |
| 2019 | Geriatric syndromes and quality of life in older adults with diabetes. | Taiwan | Community | Community-dwelling older adults | Yang et al. | To describe Geriatric syndrome (GS) and their contribution to Quality of Life (QOL) for Chinese older adults. | Primary research | No | Those who had severe cognitive impairments leading to difficulty in correctly answering simple questions were excluded. | 316 | ≥ 60 | 69.6 ± 6.6 | - | Hypertension, Cataract, Dizziness | - | Yes | Not stated | Sleep disturbance: participants were asked about the number of hours they actually slept on a typical night in the past month. Five or fewer hours was used as the cut-off. Number of over the counter or prescription sleeping medications. | Not specified |
| 2019 | Evaluation of modifiable geriatric syndromes among hip fracture patients; A pilot study | Turkey | Secondary care | Hospital inpatients | Turkmen | To plan comprehensive geriatric assessment of hospitalized hip fracture patients in Istanbul Medical School. | Conference abstract | No | - | 14 | 71-92 | 83.6 ± 5.3 | - | Visual problem, incontinence | - | Yes | Not stated | Insomnia | Social care specialist and dietician |
| 2019 | Depression and geriatric assessment in older people admitted for hip fracture | Mexico | Secondary care | Admissions for hip service | Charles-Lozoya et al. | To assess depression scores and other mental and physical health variables in older people with and without depression, admitted to a traumatology ward for a hip fracture. | Primary research | No | - |  | > 65 | - | 78 (72-83) | Hip fracture | Insomnia Severity Index Scale | No | - | - | Trained doctors |
| 2019 | Screening of geriatric syndromes in patients with falls | Russia | Secondary care | Geriatric hospital | Khovasova et al. | To assess the structure and prevalence of geriatric syndromes among people over 60 years with falls. | Conference abstract | No | - | 155 | - | 75.84 | - | - | - | Yes | Not stated | Sleep disorders | Not specified |
| 2019 | Rapid geriatric assessment iPad application: A practical way of identifying older adults at risk in primary care | Singapore | Primary care | Primary care practice | Merchant et al. | To explore the feasibility and implementation of RGA iPad application in two busy primary care practices and to determine overall prevalence of frailty,sarcopenia, and anorexia of ageing (AA). | Primary research | No | - | 2589 | ≥ 65 | 73.1 ± 6.5 | - | Sarcopenia, anorexia of aging, cognitive impairment | Patient Health Questionnaire (PHQ-9) | Yes | Not stated | Sleep apnea | Trained care coordinators and/or nurse |
| 2019 | Nocturia and its clinical implications in older women | Turkey | Secondary care | Outpatients admitted to a geriatric centre | Ünsal et al. | To demonstrate the relationship between nocturia and geriatric syndromes, and comprehensive geriatric assessment parameters (CGA) in older women. | Primary research | No | Patients who have severe illness that may impair general healthstatus, such as acute cerebrovascular event, sepsis, acute renal failure,acute coronary syndrome, and acute respiratory failure; patients under65 years of age and male; patients who did not agree to undergo the CGA; patients who had active malignancy and a history of urinary tract cancers such as renal, pelvis, and bladder cancer even if they were incomplete remission; and patients who had urinary catheters; patients who had lower urinary tract infections symptoms such as fever, lower abdomen discomfort, frequent and painful urination or who had uropathologic condition such as urinary stones. | 858 | ≥ 65 | 74.1 ± 8.2 | - | Hypertension, diabetes mellitus, osteoarthritis | Insomnia Severity Index | Yes | "Generally, during the past 30 days, how many times do you usually urinate after you have gone to sleep at night until the time you got up in the morning?" | - | Not specified |
| 2019 | 10 Playing Our Part - Are The Occupational Therapists Completing A Comprehensive Geriatric Assessment?...67th Annual & Scientific Meeting of the Irish Gerontological Society, Innovation, Advances and Excellence in Ageing, 26â€“28 September 2019, Cork, Irela | Ireland | Secondary care | Inpatients of an oncological centre | Lavery and O'Connor | To investigate whether the occupational therapists in the Medicine for the Older Person service are adhering to guidelines for CGA. | Conference poster | No | - | 10 | - | - | - | - | - | No | - | Sleep | Occupational therapists |
| 2019 | Screening for sleep apnea in very old patients admitted in a hospital cardiogeriatric unit: A pilot study | France | Secondary care | hospital cardiogeriatric unit | Ravera et al. | To investigate sleep apnea syndrome prevalence in older individuals with cardiovascular diseases. | Conference abstract | Yes | - | 43 | ≥ 70 | 85.1 ± 6.8 | - | Heart failure | Epworth sleepiness scale (ESS), the Berlin Questionnaire and NoSAS Score. | No | - | - | Not specified |
| 2019 | Integrated care for older people (ICOPE): Guidance for person-centred assessment and pathways in primary care | Switzerland | Primary care | WHO guideline | World Health Organisation | These guidelines set out 13 evidence-based recommendations for health and care workers to help develop and carry out person-centred integrated care for older people (ICOPE) at the community level. | Guideline | No | - | - | ≥ 60 | - | - | - | PHQ-9 (Patient Health Questionnaire-9) | No | - | - | Health and social care workers |
| 2019 | Disability in older adults across the continuum of cognitive decline: unique contributions of depression, sleep disturbance, cognitive deficits and medical burden. | Australia | Tertiary care | Healthy Brain Ageing (HBA) Clinic | LaMonica et al. | To investigate the association between disability, depression, and cognitive deficits relative to both established and novel correlates of functional impairment including age, medical burden, and sleep quality in a clinical sample. of older adults | Primary research | Yes | Limited English proficiency, intellectual disability, Mini-Mental State Examination (MMSE) <20, history of stroke, traumatic brain injury (with loss of consciousness >30 minutes), neurological or other medical condition known to affect cognition, current substance misuse, or major non-affective psychiatric disorder (e.g., psychosis). | 442 | 44-89 | 67.1 ± 9.3 | - | Heart disease, diabetes | Pittsburgh Sleep Quality Index | No | - | - | Not specified |
| 2019 | The prevalence and clinical risk factors of insomnia in the Chinese elderly based on comprehensive geriatric assessment in Chongqing population | China | Secondary care | Outpatients and inpatients | Zou et al. | To investigate the prevalence and clinical risk factors of insomnia in a representative sample of Chinese elderly (≥ 60 years) in Chongqing. | Primary research | Yes | i) age < 60 years old; (ii) the presence of serious physical illnesses (consciousness disturbance, hearing disorder, dystopia); and (iii) refusal to participate in the program. | 597 | ≥ 60 | - | - | Cognitive impairment, hypertension, dizziness | Pittsburgh Sleep Quality Index | No | - | - | Clinical psychiatrist and a geriatrician |
| 2019 | Comprehensive Geriatric Assessment Toolkit for Primary Care Practitioners | UK | Primary care | For use across settings | British Geriatrics Society | An introduction to Comprehensive Geriatric Assessment (CGA) in primary care settings. It is divided into two parts. Section 1 covers the basics of CGA in primary care, while Section 2 relates CGA to specific clinical presentations that may be encountered in practice. | Toolkit | No | - | - | - | - | - | - | - | Yes | Not stated | Sleep disturbance | GPs, pharmacist, nurses |
| 2020 | Characteristics of geriatric syndromes and geriatric ressources of older, multimorbid patients in 4 different settings | Germany | Primary and secondary care | Inpatient and general practice | Lena et al. | To assess the actual prevalence of geriatric syndromes and geriatric resources across settings. | Conference abstract | No | - | 950 | ≥ 65 | 77.7 ± 5.8 | - | Kidney failure, cardiovascular diseases and acute infection | - | Yes | Not stated | Insomnia | Not specified |
| 2020 | Comparison and concordance between CGA using the VALINTAN computer tool and that performed by geriatricians: Pilot study | Spain | Primary care | - | Lesende et al. | To compare/analyze the concordance in diagnoses/interventions between the VALINTAN applied by PC physicians/nurses and a CGA by geriatricians, in 12 patients aged 70 and older. | Conference abstract | No | - | 12 | ≥ 70 | - | - | - | VALINTAN (online CGA software) | Yes | Not stated | Insomnia | Physicians and nurses |
| 2020 | Comprehensive geriatric assessment in primary care practices: a multi-centered, cross-sectional study in Krakow, Poland | Poland | Primary care | General practice | Pachołek et al. | To evaluate the prevalence and severity of deficits typical of seniors and find relationships between CGA results and selected factors | Primary research | No | Life expectancy below six months and inability to communicate in Polish. | 438 | 65-96 | 75.6 ± 7.9 | - | Visual problems, hypertension, worsening of hearing | Athens Insomnia Scale | No | - | - | Trained physician |
| 2020 | Prevalence of Geriatric Syndromes and the Need for Hospice Care in Older Patients of the Emergency Department: A Study in an Asian Medical Center | Taiwan | Secondary care | Emergency Department | Ke et al. | To clarify the prevalence of geriatric syndromes and the need for hospice care in the ED in Asian populations. | Primary research | No | Patients who were suffering from acute strokes and acute myocardial infarctions or were awaiting surgery. | 693 | ≥ 65 | 78 ± 8.2 | - | Dementia, nutrition problem, pain | - | Yes | Not stated | Sleep disturbances affecting activities of daily living | Nurse practitioner or nurse |
| 2020 | The prognostic significance of geriatric syndromes and resources | Germany | Secondary care | Hospital inpatients | Meyer et al. | To ascertain the impact of common GS and GR on patients prognosis as assessed by means of the comprehensive geriatric assessment (CGA)-based Multidimensional Prognostic Index (MPI). | Primary research | No | (1) evaluation point outside of the established time window (2) inability to consent (3) inability to speak German or speech disorder (4) refusal to participate, (5) prefinal situation or death before recruitment and (6) multiple times no possibility to meet the patient for recruitment. | 135 | ≥ 70 | 78.1 ± 5.1 | 78.1 | Instability, Sensorial impairment, Instability | - | Yes | "Do you have regularly problems with sleeping?" | - | Not specified |
| 2020 | Design and methodology of the Aging Nephropathy Study (AGNES): a prospective cohort study of elderly patients with chronic kidney disease | Brazil | Tertiary care | Nephrogeriatric Clinic | Coelho et al. | To investigate clinical, biochemical and demographic factors associated with RRT initiation and mortality of patients with CKD stage 4 or 5 who are aged 70 years and older. | Primary research | No | Patients with life expectancy lower than 6 months according to the investigator's judgment; patients with untreatable cancer. | 200 | ≥ 70 | - | - | - | Pittsburgh sleep quality index (PSQI) and The Epworth Sleepiness Scale (ESS) | No | - | - | Dietitian, nurse, nephrologist, and geriatric physician |
| 2021 | Quality of Sleep and Related Factors on Elderly Residents of the Nursing Homes | Turkey | Community | Nursing home | Yurt and Cubukcu | To determine the rate of sleep disorders in elderly people aged 65 and older, living in nursing homes in Samsun, and also to investigate the factors affecting sleep quality through a comprehensive geriatric assessment. | Primary research | Yes | - | 84 | ≥ 65 | 77.1 ± 8 | - | Hypertension, heart failure, diabetes mellitus | Richards- Campbell Sleep Questionnaire (RCSQ) and Epworth Sleepiness Scale (ESS). | No | - | - | Not specified |
| 2021 | Sleep quality and sleep-disturbing factors of geriatric inpatients | Turkey | Secondary care | Geriatric inpatients in medical wards | Åžen et al. | To evaluate the change in sleep patterns and sleep quality in older patients during hospitalization. | Primary research | Yes | Non-cooperated patients who had severe cognitive impairment or delirium, severe speech/hearing disorders, terminally ill patients, and those who declined to participate. | 101 | ≥ 65 | 73.5 ± 5.2 | - | Infections, malignancy, Gastrointestinal disease | Insomnia Severity Index and Pittsburgh Sleep Quality Index | Yes | Not Stated | Sleep disturbance factors - Pain, restless legs syndrome symptoms (RLS), nightmares, delirium, fluid intake before bedtime, physical restrictions (use of oxygen, urinary catheters, etc.), daytime napping, prescribed sleeping pills, sedative medications and/or medications stimulating arousals | Not specified |
| 2021 | Assessing the impact of comprehensive geriatric assessment directed interventions in asian geriatric oncology patients | Singapore | Secondary care | Geriatric Oncology service outpatient clinic | Pang et al. | The study endpoints were i) the presence of issues of concern identified on the CGA not identified by the primary oncologists. ii) Improvement in the patient's QOL for patients provided with tailored multidimensional interventions. | Conference abstract | No | - | 230 | >70 | - | - | Cancer | EORTC QLQ-C30 | No | - | - | Primary oncologists |
| 2021 | Prevalence of sarcopenia and its effect on geriatric status in elderly patients with falls | Russia | Secondary care | - | Khovasova and Naumov | To assess the effect of sarcopenia on the geriatric status of patients over 60 years with falls. | Conference abstract | No | - | 310 | ≥ 60 | - |  |  | - | Yes | Not stated | Sleep disorders | Not specified |
| 2021 | Frailty is associated with poor sleep quality in the oldest old | Turkey | Secondary care | Geriatric outpatient clinic | Çavuşoğlu et al. | To evaluate the relationship between frailty and sleep quality in the oldest old patients | Primary research | Yes | Patients who met the Petersen criteria for mild cognitive impairment or the Diagnostic and Statistical Manual of Mental Disorders-Fifth Edition (DSM-5)criteria for dementia. patients with major depressive disorder and bipolar  disorder according to the DSM 5 criteria. Patients with disabilities (amputations, stroke-induced sequela, aphasia, and hearing problems), decompensated heart failure, acute myocardial infarction, acute stroke, exacerbation of chronic obstructive pulmonary disease, acute illnesses (i.e. infections and unstable general conditions), and those admitted to hospital or the intensive care unit within the last 3 months. | 100 | 80-92 | - | 84 (80-91) | Hypertension, urinary incontinence, diabetes mellitus | Pittsburgh Sleep Quality Index | No | - | - | Geriatrician |
| 2021 | The effects of the glycaemic control on the severity of the delirium in the advanced phase of Alzheimer's disease | Italy | Secondary care | Hospital outpatients | Martocchia et al. | To evaluate the relationship between delirium and glycemic control in the advanced phases of AD | Primary research | No | Subjects with other diseases, such as territorial infarction, intracranial hemorrhage, brain tumor, hydrocephalus, or severe white matter hyperintensities (WMH). | 38 | - | 83.3 ± 5.7 | - | Advanced Alzheimer's disease, Type 2 diabetes | Cornell Scale for Depression in Dementia | No | - | - | Not specified |
| 2021 | SARC-F Questionnaire Detects Frailty in Older Adults | Turkey | Secondary care | Geriatric outpatient clinic | Bahat et al. | To investigate the efficiency and performance of the SARC-F questionnaire to screen or evaluate frailty in older people. | Primary research | No | Being younger than 65 years old, refusal to participate, moderate to severe dementia, or other cognitive/communicative problems (e.g., severe hearing loss). | 447 | ≥ 65 | 74.5 ± 6.6 | - | Hypertension, hyperlipidemia, diabetes mellitus | - | Yes | Not Stated | Sleep disorders | Physiotherapist |
| 2021 | Associations between mild hyponatremia and geriatric syndromes in outpatient settings | Turkey | Secondary care | Geriatric outpatient clinic | Heybeli et al. | To determine associations between mild hyponatremia and results of comprehensive geriatric assessment tools in outpatient settings. | Primary research | No | Patients with dementia and Parkinson's disease; patients with psychotic diseases, patients with severe osteoarthritis or neuromuscular disease, immobile patients; and/or acute events which may alter results of geriatric assessment tools including respiratory failure, acute liver failure, sepsis, acute cerebrovascular event, acute coronary syndrome and malignancy conditions, patients who did not agree to undergo the comphrensive geriatric assessment, lack of sodium measurement, hypernatremia, moderate to severe hyponatremia or patients who had missing data in the hospital records. | 1255 | ≥ 65 | 73.7 ± 8.3 | - | Hypertension, diabetes mellitus, chronic kidney disease | Insomnia Severity Index | Yes | "Generally, during the past 30 days, how many times do you usually urinate after you have gone to sleep at night until the time you got up in the morning?"Response options included choices ranging from 0 to 3, or 4 or more per night. | - | Not specified |
| 2021 | Comprehensive geriatric assessment in the emergency department for the prediction of readmission among older patients: A 3-month follow-up study | Taiwan | Secondary care | Emergency Department | Huang et al. | To use comprehensive geriatric assessment (CGA) surveying the patterns of ED visits among older patients and determine frailty associated with the risk of revisits/readmission | Primary research | No | (1) Diagnosed with malignant tumors within 3 years who were not in a stable disease state, including the need to receive tumor-related treatment or to receive unacceptable conditions for palliative care, (2) with autoimmune diseases who were not in a stable disease state requiring immunosuppressive agents to reach therapeutic targets, (3) unable to cooperate with blood evaluation or routine physiology test, (4) unable to cooperate with long-term follow-up assessment, and (5) subjects who had been enrolled in this study | 270 | ≥ 75 | 85.1 ± 5.6 | - | - | - | Yes | Not Stated | Sleeplessness | Research nurse |
| 2021 | Prevalence and the factors associated with malnutrition risk in elderly Chinese inpatients | China | Secondary care | Hospitalised patients | Liu et al. | To investigate the prevalence of poor nutritional status and identify comprehensive geriatric assessment-based clinical factors associated with increased malnutrition risk to assessing malnutrition risk in hospitalized elderly patients in China. | Primary research | No | - |  | 60-93 | 76.4 ± 7.7 | - | Hypertension, diabetes, coronary disease | - | Yes | Not Stated | Insomnia | Geriatricians and nurses |
| 2021 | Comparative Evaluation of Predictive Ability of Comprehensive Geriatric Assessment Components Including Frailty on Long-Term Mortality | Turkey | Secondary care | Geriatric medicine outpatient clinic | Varan et al. | To compare the predictive value of all comprehensive geriatric assessment (CGA) parameters with the predictive value of frailty assessment (with Edmonton Frailty Scale (EFS) and Fried Frailty Index (FFI)) for long-term mortality in older adults. | Primary research | No | - | 967 | 65-94 | - | 73 | Hypertension, urinary incontienene, diabetes mellitus | - | Yes | How many hours did you sleep on average during the past week? | - | Not specified |
| 2021 | Promoting brain health in an integrated care outreach programme | Ireland | Community | Regional integrated care outreach programme | Carthy et al. | To review current practices and barriers to brain health guidance in a regional integrated care outreach programme (ICOP). | Poster presentation | No | - | 30 | - | 80.3 | - | Mild Cognitive Impairment | - | Yes | Not stated | Sleep disturbance (sleep satisfaction) | Not specified |
| 2022 | Assessment of older acute hip fracture patients: from the view of geriatricians | Turkey | Secondary care | Orthogeriatric assessment centre | Catikkas et al. | To report the results derived from the first two orthogeriatric assessment centers in Turkey. | Conference abstract | No | - | 103 | 63-95 | 81.2 ± 7.8 | - | Hip fracture | - | Yes | Not stated | Difficulty falling asleep and/or maintaining sleep | Geriatrician |
| 2022 | Comprehensive geriatric assessment parameters and falls risk in older adults with mild-cognitive impairment | Turkey | - | - | Soysal et al. | To investigate the risk factors for falls in older adults living with Mild Cognitive Impairment (MCI) by using Comprehensive geriatric assessment (CGA) | Conference abstract | No | - | 1596 | - | 77.5 ± 7.6 | - | Mild Cognitive Impairment | Epworth Sleepiness Scale score and Insomnia Severity Index | No | - | - | Not specified |
| 2022 | Awareness of caregivers of geriatric deficits among older people-the results of a cross-sectional study in Krakow, Poland | Poland | Primary care | Primary care practices | Pachołek et al | Comparison of patients with and without caregivers (CGs) in terms of demographic and medical issues; characterisation of the group of CGs caring for senior patients; establishing whether CGs are aware of patients' deficiencies in the areas of CGA. | Primary research | No | Bedridden seniors. | 438 | ≥ 65 | 76.8 ± 8.2 | - | - | Athens Insomnia Scale | No | - | - | Not specified |
| 2022 | A simple method for clinical implications of pain; comprehensive geriatric assessment | Turkey | Secondary care | Geriatric outpatients admitted to a university hospital | Efendioğlu et al. | To identify the factors associated with chronic pain by performing a comprehensive geriatric assessment (CGA) on geriatric outpatients. | Primary research | No | Participants with diseases that affect the assessment of muscle function and pain perception (e.g., cancer, rheumatic diseases, neuromuscular diseases, immobility, neurodegenerative diseases, neuropathy, visual and hearing disorders, and peripheral artery disease). | 225 | - | 72.9 ± 6.9 | - | Hypertension, diabetes mellitus, coronary artery disease | Pittsburgh Sleep Quality Index | No | - | - | Not specified |
| 2022 | Comprehensive Geriatric Assessment (CGA) Screening of Geriatric Patients in a Tertiary Care Hospital: a Cross-sectional Study in India | India | Tertiary care | Outpatients departments | Salagre et al. | To measure the prevalence of undiagnosed medical problems through comprehensive geriatric assessment in various outpatient departments and discuss the applicability of CGA in the Indian tertiary healthcare setup. | Primary research | No | Patients with terminal illness or critical conditions and those with known pre-existing diagnoses of severe psychiatric disorders. | 262 | ≥ 60 | 68.1 ± 5.3 | - | Depression, Low vision, Urinary incontinence | Insomnia Severity Index | No | - | - | Not specified |
| 2022 | Association between depression scores and comprehensive geriatric assessment and frailty in geriatric outpatients with somatic complaints: an observational cross-sectional study | Turkey | Secondary care | Geriatric outpatient clinic | Öztorun et al. | To reveal the importance of newly diagnosed high depression scores in the geriatric population admitted to outpatient clinics with somatic complaints. | Primary research | No | Patients who were too disabled to complete a comprehensive geriatric assessment, who had dementia with a Mini-Mental State Examination (MMSE) score below 15, or who were known to have a previous diagnosis of depression and were receiving treatment were excluded. | 235 | - | 73.6 ± 6.4 | - | Hypertension, Diabetes mellitus, COPD | - | Yes | Not Stated | Sleep duration, sleep time, sleep quality (poor, good) | Not specified |
| 2022 | Falls Among Older Adults During the COVID-19 Pandemic: A Multicenter Cross-Sectional Study in Vietnam | Vietnam | Secondary care | Outpatients geriatric clinics | Nguyen et al. | To determine the prevalence of all fall events among older outpatients during the COVID-19 pandemic in Vietnam as well as the prevalence of post- COVID-19 falls among COVID-19 infected older individuals. We also aimed to investigate the differences between the faller and non-faller groups and the COVID-19 infected and COVID-19 non-infected groups and identify the factors associated with all fall events and post-COVID-19 falls. | Primary research | No | - | 814 | ≥ 60 | 71.8 ± 7.3 | - | Hypertension, coronary artery disease, osteoarthritis | Pittsburgh Sleep Quality Index | No | - | - | Not specified |
| 2022 | Psychological Resilience Among Older Japanese Adults With Mild Cognitive Impairment During the COVID-19 Pandemic | Japan | Community | Questionnaire sent to participants | Matsumoto et al. | To investigate the impact of the COVID-19 pandemic on psychological resilience in older adults with MCI and to explore associated physical and psychosocial factors | Primary research | No | The exclusion criteria were older adults diagnosed with dementia and having a Mini-Mental State Examination (MMSE) (25) score of < 24 points at baseline; respondents who self-reported a diagnosis of depression and those who had missing data on the CD-RISC-10. | 268 | 65-85 | - | - | - | Pittsburgh Sleep Quality Index | No | - | - | Not specified |
| 2022 | Unhealthy oral status contributes to the older patients with cognitive frailty: an analysis based on a 5-year database | China | Secondary care | Hospitalized patients | Jiang et al. | To investigate the oral health status and potential risk factors of elderly hospitalized patients aged 60 years or older with cognitive frailty. | Primary research | No | (i) aged<60 years; (ii) Patients with simple cognitive impairment or physical frailty; (iii) Patients with dementia; (iv) Patients with acute oral pain. | 425 | ≥ 60 | - | - | Hypertension, coronary heart disease, diabetes | Pittsburgh Sleep Quality Index | No | - | - | Not specified |
| 2022 | The Clinical Management of Deconditioned Patient | Russia | Secondary care | Outpatient clinics | Prashchayeu et al. | To identify the clinical manifestations of subacute functional deficits. | Primary research | No | Age 64 years or less and 75 years or more, severe somatic pathology, cognitive disorders of the dementia level, oncological diseases, senile asthenia. | 172 | 65-74 | 69.2 ± 2.2 | - | Subacute functional deficit | Ten-point visual analogue scale for sleep quality evaluation and The Beck Depression Inventory (BDI) | No | - | - | Therapists |
| 2023 | Comparison of Frailty Criteria, Cognitive Function, Depressive and Insomnia Symptoms in Men with Localized and Advanced Prostate Cancer under Androgen Deprivation Therapy | Spain | Community | Community-dwelling | Mafla-España et al. | To assess whether there are differences in frailty between metastatic (mPCa) and localized prostate cancer under the effects of androgen blockade. | Primary research | No | Not being able to correctly understand the Spanish language or the content of the questions asked in the psychological and functional assessment. | 63 | - | 73.6 ± 1.2 | - | Metastatic (mPCa) and localized prostate cancer | Athens Insomnia Scale | No | - | - | Not specified |
| 2023 | Sleep quality and its associations with disease activity and quality of life in older patients with rheumatoid arthritis | Turkey | Secondary care | Rheumatology outpatient clinic | Deniz et al. | To compare sleep quality and its associations with disease activity and quality of life between older patients with rheumatoid arthritis and age- and gender-matched controls. | Primary research | Yes | History of malignancy, dementia, obstructive sleep apnea, psychiatric diseases (major depression, psychosis), neurological diseases restricting activities of daily living (e.g., Parkinson's disease, multiple sclerosis, stroke), admission to a hospital with acute illness in the previous 3 months, and unwilling to provide written informed consent. | 100 | 65-86 | - | 71 (65-86) | Rheumatoid arthritis, hypertension, diabetes mellitus | Pittsburgh Sleep Quality Index | No | - | - | Not specified |
| 2023 | Effects of continuous positive airway pressure on comprehensive geriatric assessment and cognitive function in elderly patients with obstructive sleep apnea syndrome | Italy | Secondary care | Sleep Disorder Unit of the Geriatrics Division | Condoleo et al. | To evaluate the effects of a 6-month treatment with Continuous Positive Airway Pressure (CPAP) on functional, humoral and cognitive parameters in a cohort of elderly patients with moderate to severe obstructive sleep apnea syndrome and different comorbidities. | Primary research | Yes | The presence of severe dementia or Alzheimer disease, central or mixed apnea syndrome, atrial fibrillation, prior ischemic or haemorrhagic major stroke, psychiatric comorbidities or drug therapy that may affect the cognitive function or sleep. (i.e. Selective Serotonin Reuptake Inhibitors, buspirone, ondasentron); and contraindications to nocturnal CPAP: recent facial or esophagogastric surgery, anatomical alterations or facial trauma and difficulty swallowing, evaluated by medical history. | 360 | ≥ 70 | 75.2 ± 4.3 | - | Type 2 Diabetes Mellitus, Heart Failure, Chronic Ischaemic Cardiopathy | Epworth Sleepiness Scale | Yes | Not Stated | Obstructuve sleep apnea syndrome (OSAS) diagnosis was made through a one-night home polygraphy registration using Vitalnight 7 | Not specified |
| 2023 | Factors Affect the Quality of Sleep in Elderly People with Metabolic Syndrome | Turkey | Secondary care | Internal Medicine Clinic of Malatya Training and Research Hospital. | Yaprak at al. | To examine the factors affecting sleep quality in the elderly diagnosed with metabolic syndrome (MetS). | Primary research | Yes | - | 378 | ≥ 65 | 72.05 | - | Metabolic syndrome (MetS). | Pittsburgh Sleep Quality Index | No | - | - | Not specified |
| 2023 | Potentially reversible causes of cognitive concerns | USA | Tertiary care | Centre for the Ageing Brain (CAB) | Chacko et al. | To investiage if comprehensive geriatric assessment (CGA) can facilitate identification of potentially reverible causes (PRC). | Conference abstract | No | - | 271 | - | - | - | - | - | Yes | Not Stated | Sleep disorders | Not specified |
| 2023 | Frailty in Older Adults - Early Identification and Management | Canada | Primary care | Community-based primary care setting | Government of British Columbia | To facilitate individualized assessment and provide a framework and tools to promote patient-centred strategies to manage frailty and prevent further functional decline. | Guideline | No | - | - | - | - | - | - | - | No | - | Sleep problems mentioned under Psychological Review | Not specified |
| 2023 | Analysis of the incidence of falls and related factors in elderly patients based on comprehensive geriatric assessment | China | Secondary care | Outpatients and inpatients | Xiao et al. | To evaluate the fall status of elderly patients in outpatient and inpatient departments of geriatrics by CGA and to analyze its related factors. | Primary research | No | Patients with acute critical illness, advanced disease and severe dementia. | 451 | ≥ 65 | - | - | Hypertension, Coronary heart disease, diabetes | Athens Insomnia Scale | No | - | - | Not specified |
| 2023 | Risk factors of poor sleep quality in older adults: an analysis based on comprehensive geriatric assessment | Turkey | Secondary care | Geriatric outpatient clinic of a university hospital | Ganidağli et al. | To investigate the association between sleep quality and common geriatric conditions in older adults. | Primary research | Yes | Patients who had a severe illness (i.e. malignancy, renal and hepatic failure, acute infection, inflammatory disease) and comorbidities that impair gait and balance (i.e. severe dementia, immobility, visual and hearing impairments). | 237 | - | 72.8 ± 6.9 | - | Hypertension, diabetes mellitus, coronary artery disease | Pittsburgh Sleep Quality Index | No | - | - | Not specified |
| 2023 | Poor sleep quality is an overlooked risk for geriatric syndromes in older adults with type 2 diabetes mellitus | Turkey | Secondary care | Geriatric outpatient clinic | Ganidağli and Ozturk | To investigate the impact of sleep quality on geriatric syndromes in older diabetic adults. | Primary research | Yes | Individuals who were unable to perform CGA tests due to severe dementia, immobility, visual (cataract, diabetic retinopathy, etc.) or hearing (presbycusis, otosclerosis, etc.) impairments malignancy, renal or hepatic failure, acute infections, severe asthma or chronic obstructive pulmonary disease, or inflammatory diseases (arthritis, gout, etc.) affecting cognition, balance, and movement were excluded. | 236 | - | 73 ± 6.9 | - | Hypertension, hyperlipidemia, depression | Pittsburgh Sleep Quality Index | No | - | - | Not specified |
| 2023 | The prevalence of excessive daytime sleepiness and associated factors in older diabetic patients | Turkey | Secondary care | Geriatric outpatient clinic of a university hospital | Catikkas et al. | To investigate the prevalence and associated factors of excessive daytime sleepiness. | Primary research | Yes | Patients with severe dementia/delirium or diseases affecting the hands that could potentially alter the ideal measurement of handgrip strength (HGS) (e.g. arthritis, carpal tunnel syndrome, or stroke), patients who took medication for sleep problems not to affect the assessment of sleepiness, could not undergo comprehensive geriatric assessment (CGA) (such as those who refused CGA or had hearing/visual impairment), and had missing data. | 227 | 65-97 | 78.8 | - | Hypertension, dementia, coronary artery disease | Epworth Sleepiness Scale | No | - | - | Not specified |
| 2023 | Recurrent lower urinary tract infections: more than an infection for older women | Turkey | Secondary care | Geriatric Outpatient Clinic | Ganidağli et al. | To investigate the association between recurrent Lower UTIs and geriatric syndromes based on comprehensive geriatric assessment (CGA). | Primary research | No | Patients with malignancy, renal impairment with glomerular filtration rate of <30 mL/min, chronic liver disease, acute or chronic infection, chronic inflammatory diseases, cognitive impairment, comorbidities that impair gait and balance (e.g. mobility, vision, and hearing disorders), and conditions that may affect bioelectrical impedance analysis measurements (e.g., amputation, edema, and severe fluid and electrolyte  imbalance). | 235 | ≥ 65 | 72.8 ± 6.8 | - | Hypertension, diabetes mellitus, coronary artery disease | Pittsburgh Sleep Quality Index | No | - | - | Not specified |
| 2023 | Essential steps in primary care management of older people with Type 2 diabetes: an executive summary on behalf of the European geriatric medicine society (EuGMS) and the European diabetes working party for older people (EDWPOP) collaboration. | EU states | Primary care | Older community-based adults with diabetes managed by their primary care team | Bourdel-Marchasson et al. | To improve standards of diabetes care of older community-based adults with diabetes managed by their primary care team. | Guideline | No | - | - | ≥ 65 | - | - | Type 2 diabetes | - | Yes | Not stated | Sleep problems | Not specified |
| 2023 | The risk of malnutrition and its clinical implications in older patients with cancer | Turkey | Secondary care | Oncology outpatient clinic | Isleyen et al. | To determine clinical importance of malnutrition risk and its clinical implications on geriatric syndromes and mortality in geriatric oncology practice. | Primary research | No | Patients with severe vision and hearing impairment that prevent communication and understanding commands during the examination, or had acute cerebrovascular event, sepsis, acute renal failure, and acute respiratory failure, as well as those with missing data in file records, patients with dementia or delirium. | 180 | ≥ 65 | 73 ± 5.6 | - | Diabetes Mellitus, Hypertension,Coronary artery disease | Epworth Sleepiness Scale | No | - | - | Geriatrician |
| 2023 | Assessment of clinical features and coexisting geriatric syndromes in newly diagnosed dementia with Lewy bodies: a retrospective study in a tertiary geriatrics setting in Turkey | Turkey | Tertiary care | Tertiary geriatric outpatient clinic | Naharci et al. | To examine the clinical features and coexisting geriatric syndromes of patients with newly diagnosed dementia with Lewy bodies. | Primary research | No | Participants with a diagnosis of other dementias, delirium, psychotic disorders, missing data, or criteria not fulfilling probable or possible dementia with Lewy bodies were excluded. | 116 | ≥ 60 | 79 ± 6.9 | - | Dementia | - | Yes | Possible REM sleep disorder was asked to family members or caregivers with the following question: have you ever seen the patient appear to act out his/her dreams while sleeping? (punched or flailed arms in the air, shouted,or screamed) .We regarded participants as having possible REM sleep disorders if they answered "yes". | - | Not specified |
| 2023 | Functional loss and worsening geriatric assessment parameters are more common in dementia with Lewy bodies than Alzheimer's disease | Turkey | Secondary care | Outpatient geriatric clinic | Soysal et al. | To compare older patients with Alzheimers disease (AD) to those with dementia with Lewy bodies (DLB) according to their dependency in daily living activities and comprehensive geriatric assessment parameters. | Primary research | No | Patients who had severe illness that may impair their general health status, such as acute cerebrovascular event, sepsis, acute renal failure, acute coronary syndrome, and acute respiratory failure, and those who did not agree to undergo the comprehensive geriatric assessment, or who had severe vision and hearing impairment that prevented communication and understanding commands during the examination. Except for those with probable Lewy Body Dementia or Alzheimer's Dementia, the patients with mild cognitive impairment were also excluded from the study. Moreover,other types of dementia were excluded, such as vascular dementia, frontotemporal dementia, and Parkinson's disease dementia. | 350 | - | 82.3 ± 5.5 | - | Hypertension, diabetes milletus, coronary artery disease | Insomnia Severity Index and Epworth Sleepiness Scale | No | - | - | Not specified |
| 2023 | How comprehensive is our Comprehensive Geriatric Assessment in clinical practice? | Ireland | Primary care | Integrated Care Teams | Dennehy et al. | to examine the specific content of CGA that are being delivered across integrated care teams in Ireland. | Primary research | No | - | 16 | - | - | - | - | - | Yes | Not Stated | Sleep | Not specified |
| 2023 | FRAIL Scale: an independent predictor of in-hospital mortality among older adults. | Turkey | Secondary care | - | Tufan et al. | To investigate the effect of geriatric syndromes on mortality in patients over 65 years of age who were hospitalized. | Primary research | No | Individuals who did not give informed consent and were hospitalized for less than 72 hours. | 85 | 66-97 | 75.9 ±7.6 | - | - | - | Yes | Not stated | Sleep disorders | Not specified |
| 2023 | Using Comprehensive Geriatric Assessment in Identifying Care Goals and Referral Services in a Frailty Intervention Clinic | Canada | Secondary care | Frailty intervention clinic (outpatients) | Aggarwal et al. | (1) as identified by the CGA, to identify the priority needs and goals of frail older adults and frail middle-aged adults who were referred to Providence Healthcare's Frailty Intervention Team (FIT) clinic (Toronto, Ontario); and (2) to identify the services to which Providence Healthcare's FIT referred these patients. | Primary research | No | - | 305 | 50-104 | 82 ±11 | - | Hypertension, dementia, Type 2 diabetes | - | No | - | "Sleep" part of mental function assessment. | Multidisciplinary team |
| 2023 | The prevalence and clinical significance of loss of appetite in older patients with chronic kidney disease | Turkey | Secondary care | Outpatient clinic | Yildiz et al. | To determine prevalence and associated factors of loss of appetite in older adults with CKD. | Primary research | No | Patients with dementia, lack of appetite assessment because of communication problems, such as hearing impairment, or refusing to undergo/complete CGA), lack of serum creatinine, stage 5 CKD (eGFR<15 ml/min), and presence of acute problems. | 398 | ≥ 60 | 80 ± 7 | - | Hypertension, Diabetes mellitus, coronary heart disease | Insomnia severity index | No | - | - | Not specified |
| 2023 | Hypertension, sleep quality, depression, and cognitive function in elderly: A cross-sectional study | China | Secondary care | Hospital Comprehensive Geriatric Assessment Database. | Chen et al. | To investigate whether sleep quality and depression have a mediating role in the association between hypertension and cognitive impairment. | Primary research | Yes | (1) brain tumors or mental illnesses including schizophrenia, organic psychosis and anxiety, (2) medical records were incomplete. | 827 | ≥ 50 | 77.2 ± 10.8 | - | Hypertension, hyperlipidemia, stroke | Pittsburgh Sleep Quality Index | No | - | - | Not specified |
| 2023 | Older Adults Service Specifications | UK | Community | Older Adults Service, for the population of Lincolnshire East Locality patients | NHS Lincolnshire Integrated Care Board (ICB) | To define the standards for the delivery of the Older Adults Service, for the population of Lincolnshire East Locality patients. | Service specification | No | People not registered with a ‘participating’ GP practice within the East locality of Lincolnshire ICB. | - | ≥ 65 | - | - | - | - | Yes | Not stated | "Sleep patterns" as subjective assessment mentioned in part of medical domains of CGA. | Multidisciplinary team |
| 2024 | Project on Geriatric Assessment in Nephrology Unit: Evaluation of Frailty and Vulnerability, Physical Capacity, and Kidney Function | Spain | Secondary care | Nephrology Unit | Lavilla | Improve geriatric assessment in Nephrology Unit. | Conference abstract | No | - | 506 | ≥ 65 | - | - | Chronic Kidney Disease | - | Yes | Not stated | Evaluation of subjective well-being (SWB) (1 to 10) and experience on sleep. | Not specified |
| 2024 | Sleep quality of vulnerable elderly people: associated factors | Brazil | Primary care | Family Health Units | Santos-Orlandi et al. | To identify factors associated with poor sleep quality in elderly dependent individuals in social vulnerability. | Primary research | Yes | Presenting hearing and/or speech deficits (stuttering, aphasia, and dysarthria) that could hinder the application and interpretation of the proposed instrument's results. | 59 | ≥ 60 | - | - | - | Pittsburgh Sleep Quality Index | No | - | - | Not specified |
| 2024 | Living Well, Ageing Well - Gloucestershire Interventions framework (frailty) | UK | - | NHS Gloucestershire ICB | NHS Gloucestershire ICB | The Gloucestershire Interventions Framework is a list of evidence based interventions and activities that facilitate a systematic and consistent approach to supporting people at risk or living with frailty. It has been adapted to incorporate Gloucestershire priorities. | Guideline | No | - | - | - | - | - | - | - | No | - | Sleeping/Insomnia mentioned in CGA guidance | Not specified |
| 2024 | Impact of Sleep Quality on Disease Activity and Clinical Outcomes in Older Patients with Inflammatory Bowel Disease | Turkey | Secondary care | Outpatient clinics | Çavuşoğlu et al. | To investigate the relationship between sleep quality, geriatric syndrome, and IBD disease activity in older patients. | Primary research | Yes | Individuals who were excluded from the study were those with unstable general illnesses, psychiatric disorders, and drug use that had the potential to impact sleep. | 91 | 50-80 | - | 60 | Inflammatory Bowel Disease | Pittsburgh Sleep Quality Index | No | - | - | Not specified |
| 2024 | TOward a comPrehensive supportive Care intervention for Older men with metastatic Prostate cancer (TOPCOP3): A pilot randomized controlled trial and process evaluation | Canada | Secondary care | Cancer centre | Alibhai et al. | Aim 1: To obtain preliminary efficacy estimates of the proposed interventions (i.e., RSM, GA + M and RSM, and GA + M) versus usual care on co-primary outcomes (a) grade 3-5 toxicity, (b) quality of life.  Aim 2: To determine preliminary efficacy and variability estimates of the proposed interventions on secondary outcomes: (a) early treatment discontinuation; (b) oncologic treatment modifications (GA arms only);  (c) instrumental activities of daily living (IADL, those needed to function independently in the community such as shopping, cooking, or using transportation); (d) symptom burden; (e) fatigue; (f) depression; (g) unplanned health care use.  Aim 3: To assess process evaluation metrics related to feasibility, acceptability, fidelity, and implementation barriers (which will inform the design and implementation of a larger RCT). | Primary research | No | Unable to speak English (as contact with the nurse interventionist is via phone, not all outcome measures are available in multiple languages); major neuropsychiatric abnormalities (e.g., severe depression or moderate-severe dementia); life expectancy <3 months as estimated by the oncologist. | 140 | ≥ 70 | - | - | Metastatic Prostate Cancer | Insomnia Severity Index | No | - | - | Trained nurse and geriatrician |
| 2024 | Prevalence and co-incidence of geriatric syndromes according to the ECOG performance status in older cancer patients | Turkey | Secondary care | Outpatient clinic | Topcu et al. | To determine the prevalence and relationship of geriatric syndromes according to the ECOG-PS in older cancer patients | Primary research | No | The patients with Eastern Cooperative Oncology Group Performance Status ≥3, those under the age of 65years, those with dementia or delirium, those with vision and hearing impairments that control understanding commands during the evaluation, and those with uncontrolled comorbid disorders were excluded. | 218 | - | 73 ± 5.6 | - | Cancer, diabetes, hyperlipidemia | Insomnia Severity Index score and Epworth Sleepiness Scale | No | - | - | Not specified |
| 2024 | Fear of falling and associated factors in older adults with heart failure | Turkey | Secondary care | Hospital cardiology department | Unes et al. | To evaluate the prevalence of fear of falling and associated factors in older adults with heart failure. | Primary research | No | (i) age <65 years; (ii) HF with preserved ejection fraction; (iii) severe dementia; (iv) terminal stage disease; (v) inability to communicate due to severe vision and hearing impairment or advanced dementia; (vi) inability to provide written informed consent; and (vii) receiving medical treatment for insomnia. | 100 | 65-90 | 72 | 72 (65-90) | Coronary artery disease, hypertension, diabetes mellitus | Epworth Sleepiness Scale | No | - | - | Not specified |
| 2024 | Comprehensive Geriatric Assessment of Rural Elderly of Odisha-Results from a Community-based Survey | India | Primary care | Community health centre | Roy et al. | To assess the fall risk, frailty, and activity of daily living and factors associated with it among the geriatric population. | Primary research | No | The bedridden patients or patients having any severe mental health disorder who were not capable of communicating during the interview were excluded from the study. | 188 | ≥ 60 | - | - | - | - | Yes | Not stated | Sleep duration | Not specified |
| 2024 | Targeting Aging and Longevity with Exogenous Nucleotides (TALENTs): Rationale, Design, and Baseline Characteristics from a Randomized Controlled Trial in Older Adults. | China | Community | Community residents | Wang et al. | To explore whether nucleotides have anti-aging effects and explore the possible pathways of the anti-aging effects of nucleotides. | Primary research | No | (1) patients with related confirmed diseases, such as autoimmune system diseases, serious cardiovascular and cerebrovascular diseases, major organ complications such as in the liver and kidney, or complicated with other serious diseases such as malignant tumors, pancreatic diseases and mental diseases; (2) having abnormal screening laboratory test values or other lab test results that would preclude study participation in the judgment of the investigator; (3) severe visual or hearing loss affecting communication; (4) being participants in other clinical trials within the last 6 months; (5) having used food or medicine that is relevant to the function being tested. | 122 | 60-70 | 65.7 ± 2.6 | - | Cardiovascular disease, renal disease, fatty liver | Pittsburgh Sleep Quality Index | No | - | - | Research staff |
| 2024 | Multiple geriatric syndromes in community-dwelling older adults in China | China | Community | Community dwelling | Wang et al. | To assess the prevalence of geriatric syndrome and identify multiple geriatric syndrome-related factors among older adults in China. | Primary research | No | Mental disorders (Alzheimer's disease, Schizophrenia), severe and end-stage diseases (expected life<12 months, that with an established life-limiting condition or in receipt of end of life palliative care services). | 706 | ≥ 60 | 70.7 ±7.4 | - | - | - | Yes | Participants were asked about the number of hours they actually slept on a typical night in the past month. | Sleep disturbance | Research nurse |
| 2024 | Risk Factors for Postoperative Cognitive Decline After Orthopedic Surgery in Elderly Chinese Patients: A Retrospective Cohort Study | China | Secondary care | Orthopaedics | Li et al. | To identify the risk factors for postoperative cognitive decline (POCD) by evaluating the outcomes from preoperative comprehensive geriatric assessment (CGA) and intraoperative anesthetic interventions. | Primary research | No | Patients with history of dementia. | 119 | ≥75 | - | 80 | Hypertension, diabetes, ischaemic heart disease | STOP- Bang questionniare | No | - | - | Orthopedists, anesthetists, neurologists, geriatricians, nutritionists, pharmacists and nurses |
| 2024 | Association of Urinary Incontinence With Depression, Insomnia, Frailty and Falls in Older Adults: a Cross-sectional Comprehensive Geriatric AssessmentA1 - Anonymous. | Turkey | Secondary care | Outpatient geriatric clinic | Demircan | To provide a holistic analysis of the geriatric syndromes associated with urinary incontinence, which can result in significant adverse outcomes in the elderly, and to explore the gender-specific relationships within these associations. | Clinical trial protocol | Yes | Dementia, those who experienced cerebrovascular occlusion with resulting neuromuscular paralysis, active infections, schizoaffective disorder, epilepsy, neuromuscular disorders,those who were wheelchair or bed-bound, advanced chronic obstructive pulmonary disease (COPD) or advanced chronic heart failure (CHF). | 635 | - | - | - | Urinary Incontinence | - | Yes | The patient was asked if they had experienced complaints of insomnia in the last 1 months. if answer is yes, considered insomnia. | - | Not specified |
| 2025 | Sleep hygiene education in older adults: effectiveness and association with comprehensive geriatric assessment | Turkey | Secondary care | Geriatric medicine outpatient clinic | Hafızoğlu et al. | This study aimed to evaluate the results of sleep hygiene education via a comprehensive geriatric assessment. | Primary research | Yes | Having moderate-severe dementia, being depressed or receiving treatment for depression, having an organic psycho-emotional state or organic degenerative disorder, using sleeping pills or drugs that disrupt the sleep cycle, and having uncontrolled medical problems that could cause underlying sleep disorders. | 141 | 65-87 | - | 70.5 (65-87) | Hypertension, hyperlipidemia, diabetes mellitus | Pittsburgh Sleep Quality Index, Sleep Hygiene Index, Epworth Sleepiness Scale, Jenkins Sleep Scale | No | - | - | Not specified |
| 2025 | Geriatrics Pocketbook | Canada | - | Geriatrics Pocketbook | National Geriatrics Interest Group | To highlight key topics in the field of geriatrics. | Geriatrics-specific resource for medical students | No | - | - | - | - | - | - | - | No | - | Geriatric review of systems (e.g. sleep). | Not specified |
| 2025 | Sleep quality, daytime sleepiness, and risk of falling: results from an exploratory cross-sectional study | Italy | Secondary care | Geriatric Service of University Hospital | Salis et al. | To investigate whether sleep quality and daytime sleepiness are associated with the risk of falls in older adults. | Primary research | Yes | Patients who failed to complete the comprehensive geriatric assessment, including the sleep evaluation for any reason, those with missing data, or those who did not provide informed consent. | 206 | ≥75 | - | 83 (79-86) | - | Epworth Sleepiness Scale and Pittsburgh Sleep Quality Index | No | - | - | Not specified |
| 2025 | Quality Standards for Memory Services 9th Edition | UK | Community | Memory Services Quality Standards | Royal College of Psychiatrists | The standards have been developed for the purposes of review and accreditation as part of the Memory Services National Accreditation Programme. | Quality standards | No | - | - | - | - | - | - | - | Yes | Not stated | Lifestyle factors e.g. sleeping patterns | Multidisciplinary team |
| 2025 | The disease burden of earlier and late-onset rheumatoid arthritis on depression and related geriatric syndromes. | Turkey | Tertiary care | Tertiary rheumatology clinic | Demircan and Kahveci | To compare the prevalence of depression and related geriatric syndromes in earlier-onset rheumatoid arthritis (EORA) patients, who have experienced prolonged inflammation and medication use, with those with late-onset rheumatoid arthritis (LORA) patients, who often present with an acute and severe course. | Primary research | No | Patients under the age of 60, those with dementia, schizophrenia, epilepsy, or similar neuropsychiatric conditions, as well as those who were bedridden or used a wheelchair. The patients with concomitant malignancy, infection,and cerebrovascular disease were also excluded. | 129 | ≥ 60 | 67.4 ± 5.2 | - | - | Insomnia Severity Index | No | - | - | Not specified |
| 2025 | Assessing the impact of sleep quality on physical function in Chinese older inpatient | China | Secondary care | Older patients admitted to internal wards | Chang et al. | Understanding how sleep quality relates to physical function to inform healthcare practices and improve outcomes. | Primary research | Yes | (1) unable or unwilling to grant informed consent  (2) inability to complete any of the performancebased tests  (3) unable to communicate with the study staff (4) presented with serious illness or terminal illness interfering with the conduct of the study or interpretation of the results  (5) having significant cognitive impairment or dementia, that could potentially interfere in assessment. | 545 | 60 - 98 | 78.4 ± 8.2 | - | Tumors, chronic renal failure, chronic heart failure. | Athens Insomnia Scale | No | - | - | Not specified |
| 2025 | The impact of adverse childhood experiences on older adults' health measured via comprehensive geriatric assessment | Turkey | Secondary care | Outpatient clinic | Ipar et al | To investigate associations between ACEs and health outcomes in older adults. | Primary research | No | 1: patients with dementia 2: severe visual or hearing impairment that prevents communication and understanding commands during the examination 3: refusal to participate in the examination 4: fatal illness, life-threatening illness in the last 6 months, or those who have been hospitalised for major surgery 5: the presence of an acute health problem (such as infection, acute kidney failure, delirium, stroke). | 276 | - | 77.9 ± 7.3 | - | Hypertension, Diabetes, Osteoarthritis | Epworth Sleepiness Scale | No | - | - | Not specified |
| 2025 | Clinical implication of anemia in older patients with dementia with Lewy bodies | Turkey | Secondary care | Single geriatric outpatient clinic | Karismaz et al . | To investigate the possible connection between anemia and various parameters of comprehensive geriatric assessment in elderly individuals diagnosed with Dementia with Lewy Bodies (DLB). | Primary research | No | Participants with critical health issues that could affect their general health status, such as acute strokes, blood infections, sudden kidney malfunction, urgent heart problems, and severe breathing difficulties. Furthermore, individuals who opted out of the CGA described in the following section were also excluded from the study. Furthermore, patients with significant visual or auditory impairments that hindered their ability to communicate effectively or comprehend instructions during the evaluation were also excluded from the study. The research excluded participants with active bleeding, including severe urinary, rectal, or gastrointestinal blood loss, as well as internal abdominal hemorrhage. Those with moderate to severe dementia or whose clinical condition (such as delirium) prevented them from undergoing CGA were also omitted. Additionally, the study did not include various forms of dementia, such as AD, vascular dementia, frontotemporal dementia, and Parkinson's disease dementia. | 147 | ≥ 65 | 85.4 ± 7.1 | - | Diabete Mellitus, hypertension, Chronic Kidney Disease | Insomnia Severity Index and Epworth Sleepiness Scale | No | - | - | Geriatrician, a psychologist, and a gerontologist. |
| 2025 | Associating factors of cognitive frailty among older people with chronic heart failure: Based on LASSO-logistic regression | China | Secondary care | Cardiology department | Gou et al. | To identify factors associated with cognitive frailty among older CHF patients in China. | Primary research | No | - | 421 | 60-90 | - | - | Chronic heart failure | Pittsburgh Sleep Quality Index | No | - | - | Researcher |
| 2025 | Development and validation of a nomogram for predicting cognitive frailty in patients on cancer | China | Tertiary care | Grade-A tertiary hospital | Wang et al. | To investigate the current status of cognitive frailty in older cancer patients and construct a risk prediction model for cognitive frailty in older cancer patients. | Primary research | No | (1) Individuals with communication difficulties (2) Individuals with symptoms of dementia or severe mental illness. | 308 | ≥ 60 | - | - | Cancer | Pittsburgh Sleep Quality Index | No | - | - | Not specified |
| 2025 | Risk Factors for Frailty in Iranian Older Adult Outpatients: a Cross-Sectional Study | Iran | Secondary care | Geriatric outpatient clinic | Mirzadeh et al. | To investigate the relationships between frailty and gender, age, and geriatric syndromes in older outpatients in geriatric clinics and various community health centers. | Primary research | No | Older adults with AMT score<8 (Bakhtiyari et al., 2014) and unable to fulfill CGA criteria (i.e., could not talk or hear) were excluded. | 364 | ≥ 60 | - | - | - | Pittsburgh Sleep Quality Index | No | - | - | Not specified |
| 2025 | Implementation of a multidisciplinary approach to care for people with HIV aged 80 years and over | UK | Secondary care | IV/geriatric hospital clinic (inpatients and outpatients) | Varadarajan et al. | To present the impact of a modified CGA in adultswith HIV aged 80 years or older, as there is a paucity ofdata in this population. | Primary research | No | - | 63 | 80-86 | 73 ± 7 | 82 (80-86) | HIV, HTN, cardiovascular disease | - | Yes | Not stated | Concerns regarding mood and sleep | Physician |
| 2025 | Implementation of a Multi-Disciplinary Geriatric Oncology Clinic in Toronto, Canada | Canada | Secondary care | Geriatric oncology clinic (outpatients) | Menjak et al. | To assess the implementation of a geriatric oncology (GO) clinic that employs CGA and determine patient outcomes. | Primary research | No | - | 100 | 63-97 | - | 80 (63-97) | Breast Cancer | - | Yes | Not stated | Sleep disturbance | Trainee physician |
| 2025 | Implications of comprehensive geriatric assessment and Traditional Chinese Medicine constitution types for integrative geriatric care | Taiwan | Secondary care | Medical center | Lin et al. | To investigate the link between Traditional Chinese Medicine (TCM) body constitutions and comprehensive geriatric assessment scores in older adults. | Primary research | No | 1) were diagnosed with cancer, end-stage renal disease, or receiving dialysis treatment, 2) had acute impaired consciousness or cognitive dysfunction, such as delirium or dementia, 3) had a history of psychiatric disorders such as depression, bipolar disorder, or schizophrenia, 4) lost the ability to walk due to other illnesses, or had difficulty communicating through speech or writing, 5)had a life expectancy of less than six months due to end-stage illnesses or cancer, and 6) received traditional Chinese medicine constitution adjustment for more than three months . | 100 | - | 74.8 ± 6.8 (Normal constitutions); 77.9 ± 6.9 (Abnormal constitutions) | - | - | - | Yes | Not stated | Sleep problems | Physician |
| 2025 | Pilot Study for Feasibility of Onco-Geriatric Intervention Model in Older Patients with Cancer in a Tertiary Academic Hospital | South Korea | Secondary care | Seoul National University Bundang Hospital | Kim et al. | To evaluate the feasibility of a tailored oncogeriatric intervention model incorporating the KG-7 screening tool. | Primary research | No | Patients younger than 70 years, those diagnosed with non-cancer conditions, and those who did not exhibit the geriatric phenotype to need further GA and geriatric intervention, despite a KG-7 score ≥ 5 | 30 | ≥70 | - | 79.5 (71-87) | Colon cancer, pancreatic cancer, gastric cancer. | - | Yes | Not stated | Insomnia (subjective discomfort reported) | Not specified |

## Appendix 3: A graph to show countries represented in scoping review

## Appendix 4: Excluded publications

[1-62]

1. Nassani NA, Ghanem S, Kassouf E, El Osta L, El Karak F, Ghosn M. The ability of physical performance test and Karnofsky performance status to identify elderly cancer patients requiring a comprehensive geriatric assessment: A comparative study. Journal of Clinical Oncology. 2013;31(15 SUPPL. 1).

2. Netzer NC, Ancoli Israel S, Frohnhofen H, Danker Hopfe H, Raschke F, Popp R, Pramsohler S. Adaptation of the Berlin questionnaire for geriatric assessment , for the international geriatric sleep medicine task force and the geriatric and diagnostic assemblies of the German sleep society. Pneumologie. 2017;71(4).

3. Cakirca M, Soysal P, Koc Okudur S, Smith L, Kilic N, Kiskac M. Anticholinergic Burden and Increased Excessive Daytime Sleepiness in Older Women. Journal of the American Medical Directors Association. 2022;23(6):1092EP-3.

4. Beck-Little R, Weinrich SP. Assessment and management of sleep disorders in the elderly. Journal of gerontological nursing. 1998;24(4):21EP-9.

5. Frohnhofen H, Popp R, Stieglitz S, Netzer N, Danker-Hopfe H. Assessment of sleep and sleep disorders in geriatric patients. Zeitschrift fur Gerontologie und Geriatrie. 2020;53(2):100-4.

6. Al-Tal S, Alkhatib B, Agraib LM, Peixoto B. The Association Between Nutritional Status, Diet Quality, and Sleep Quality Among the Elderly in Jordan: A Cross‐Sectional Study. Journal of Aging Research. 2025:1-9.

7. De la OMA, Torres AC, Ramos-Lopez WA, Covarrubias-Gomez A, Quiroz P, Sanchez-Roman S, et al. Association of pain with the presence of additional supportive care (SC) needs in patients with advanced cancer. Journal of Clinical Oncology. 2022;40(16 Supplement 1).

8. Auyeung TW, Lee JSW, Leung J, Kwok T, Leung PC, Woo J, Wing YK. Cognitive deficit is associated with phase advance of sleep-wake rhythm, daily napping, and prolonged sleep duration--a cross-sectional study in 2,947 community-dwelling older adults. Age (Dordrecht, Netherlands). 2013;35(2):479-86.

9. Banerjee D, Mukherjee S, Paul N. Comprehensive Geriatric Assessment (CGA) for the psychiatrists: A ONE-STOP TOOL for assessing older adults? Indian Journal of Psychiatry. 2020;62(7 Supplement 1):S156EP-S7.

10. Palicio C, Boque C, Antonio M, Bido M. Comprehensive geriatric assessment and intervention proposal in an outpatient hematology ward. European Geriatric Medicine. 2017;8(Supplement 1):S74.

11. Xue D, Han S, Jiang S, Sun H, Chen Y, Li Y, et al. Comprehensive geriatric assessment and traditional Chinese medicine intervention benefit symptom control in elderly patients with advanced non-small cell lung cancer. Medical Oncology. 2015;32(4):1EP-7.

12. Yoo JW, Nakagawa S, Kim S. Delirium and transition to a nursing home of hospitalized older adults: a controlled trial of assessing the interdisciplinary team-based "geriatric" care and care coordination by non-geriatrics specialist physicians. Geriatrics & gerontology international. 2013;13(2):342-50.

13. D'Onofrio G, Panza F, Sancarlo D, Paris FF, Cascavilla L, Mangiacotti A, et al. Delusions in patients with Alzheimer's disease: A multidimensional approach. Journal of Alzheimer's Disease. 2016;51(2):427EP-37.

14. Elder GJ, Colloby SJ, Lett DJ, O'Brien JT, Anderson KN, Burn DJ, et al. Depressive symptoms are associated with daytime sleepiness and subjective sleep quality in dementia with Lewy bodies. International journal of geriatric psychiatry. 2016;31(7):765-70.

15. Development and Validation of a Protected Discharge Model Using Technology in Multimorbidity and Poly-treated Older Subjects. The PRO-HOME StudyA1 - Anonymous. clinicaltrialsgov. 2023.

16. Guner M, Bas AO, Ceylan S, Kahyaoglu Z, Coteli S, Unsal P, et al. Dysphagia is closely related to frailty in mild-to-moderate Alzheimer's disease. BMC geriatrics. 2023;23(1):304.

17. Bouman A, Van Rossum E, Ambergen T, Kempen G, Knipschild P. Effects of a home visiting program for older people with poor health status: A randomized, clinical trial in the Netherlands. Journal of the American Geriatrics Society. 2008;56(3):397EP-404.

18. Akhtar OS, Wang C, Attwood K, Carozza D, Gage-Bouchard ER, Kaijar T, et al. Evaluating the role of baseline geriatric assessment in predicting quality of life in older adults with non-Hodgkin lymphoma on oral targeted therapies. Blood. 2021;138(SUPPL 1):4094.

19. Hamilton C, Christmas C. Evaluation and Management of Difficult Symptoms in Older Adults in Primary Care. The Medical clinics of North America. 2020;104(5):885-94.

20. Wald HL, Glasheen JJ, Guerrasio J, Youngwerth JM, Cumbler EU. Evaluation of a hospitalist-run acute care for the elderly service. Journal of Hospital Medicine. 2011;6(6):313EP-21.

21. Bloom HG, Ahmed I, Alessi CA, Ancoli-Israel S, Buysse DJ, Kryger MH, et al. Evidence-based recommendations for the assessment and management of sleep disorders in older persons. Journal of the American Geriatrics Society. 2009;57(5):761-89.

22. Koc Okudur S, Soysal P. Excessive Daytime Sleepiness is Associated With Malnutrition, Dysphagia, and Vitamin D Deficiency in Older Adults. Journal of the American Medical Directors Association. 2021;22(10):2134EP-9.

23. Five-step approach targets insomnia in older adults. Geriatrics. 2005;60(8):13-.

24. Morris GS, des Bordes JKA, Holmes HM, Giralt S. Frailty and stem cell transplantation in the older patient with cancer. Journal of Geriatric Oncology. 2012;3(3):273EP-8.

25. Frailty in Community Dwelling Older Adults With Heart FailureA1 - Anonymous. clinicaltrialsgov. 2019.

26. Tran HPT, Leonard SD. Geriatric Assessment for Primary Care Providers. Primary Care - Clinics in Office Practice. 2017;44(3):399EP-411.

27. Rafters SL, Schleper J, Lean N, Hwang M, Rohra A, Lillington L. Geriatric Syndrome Screening: Implementing an Evidence-Based Process in Hospitalized Older Adults With Cancer. Clinical journal of oncology nursing. 2021;25(3):297EP-304.

28. Naeim A, Reuben D. Geriatric syndromes and assessment in older cancer patients. Oncology (Williston Park, NY). 2001;15(12):1567EP-91.

29. Fulmer T. How to try this. Fulmer SPICES: a framework of six 'marker conditions' can help focus assessment of hospitalized older patients. AJN American Journal of Nursing. 2007;107(10):40-9.

30. Liu R, Shao W, Lai JKL, Zhou L, Ren M, Sun N. Identification of comprehensive geriatric assessment-based risk factors for insomnia in elderly Chinese hospitalized patients. Aging Medicine. 2021;4(1):26EP-34.

31. Pickens AW, Forest DJ, Wyderski RJ, Williams JA, Huang KE, Hazzard WR, et al. Identifying Risk of Sleep Apnea and Major Hospital Events in an Older Inpatient Population. Journal of the American Geriatrics Society. 2018;66(9):1847-8.

32. Gregersen M, Jensen K, Orum M. The impact of multidisciplinary geriatric follow-up on quality of life in older, non-surgical prefrail and frail patients with cancer - A randomized controlled trial. Journal of Geriatric Oncology. 2024;15(8):102069.

33. Avidan AY. Insomnia in the geriatric patient. Clinical Cornerstone. 2003;5(3):51-60.

34. Sween RR, Grey C. Interprofessional geriatric consultation to improve vulnerable acute care patient outcomes. Journal of the American Geriatrics Society. 2017;65(Supplement 1):S207.

35. Lee C, Park YH, Cho B. Leveraging network analysis to determine sex differences in factors associated with frailty among older adults living alone. BMC geriatrics. 2023;23(1):38.

36. Akahane M, Yoshihara S, Maeyashiki A, Tanaka Y, Imamura T. Lifestyle factors are significantly associated with the locomotive syndrome: a cross-sectional study. BMC geriatrics. 2017;17(1):241.

37. (MIGA) Mini Geriatric Assessment as Compared to the Current CGAA1 - Anonymous. clinicaltrialsgov. 2011.

38. Kirkhus L, Harneshaug M, Saltyte Benth J, Gronberg BH, Rostoft S, Bergh S, et al. Modifiable factors affecting older patients' quality of life and physical function during cancer treatment. Journal of Geriatric Oncology. 2019;10(6):904EP-12.

39. D'Onofrio G, Sancarlo D, Panza F, Copetti M, Cascavilla L, Paris F, et al. Neuropsychiatric symptoms and functional status in alzheimer's disease and vascular dementia patients. Current Alzheimer Research. 2012;9(6):759EP-71.

40. Gilsenan I. NURSING INTERVENTIONS TO ALLEVIATE INSOMNIA. Nursing Older People. 2012;24(4):14-8.

41. Edelstein BA, Woodhead EL, Segal DL, Heisel MJ, Bower EH, Lowery AJ, Stoner SA. Older adult psychological assessment: current instrument status and related considerations. Clinical Gerontologist. 2008;31(4):1-35.

42. Optimization of Older Adult Allogeneic Hematopoietic Cell Transplant Candidates to Improve Survival (OTIS)A1 - Anonymous. clinicaltrialsgov. 2022.

43. Culakova E, Mohile S, Peppone L, Ramsdale E, Maggiore R, Patil A, et al. PATIENT-REPORTED SYMPTOM BURDEN AND ASSOCIATION OF GERIATRIC ASSESSMENT (GA) IMPAIRMENTS WITH THE SYMPTOM BURDEN IN OLDER ADULTS WITH ADVANCED CANCER RECEIVING SYSTEMIC TREATMENT. Journal of Geriatric Oncology. 2019;10(6 Supplement 1):S68EP-S9.

44. Woods JD, Zeidner JF, Van Deventer HW, Jamieson K, Matson M, Zhang J, et al. Phase Ib trial of lenalidomide as post-remission therapy for older adults with acute myeloid leukemia: Safety and longitudinal assessment of geriatric functional domains. Journal of Geriatric Oncology. 2022;13(4):499EP-504.

45. Garfinkel D. Poly-de-prescribing to treat polypharmacy: efficacy and safety. Therapeutic Advances in Drug Safety. 2018;9(1):25EP-43.

46. Sayed SA, Hamza SA, Adly NN, Tawfik HM. Prevalence of Cognitive Impairment in Post Stroke Patients. QJM: An International Journal of Medicine. 2024;117(Supplement 2):ii139EP-ii40.

47. Martin JL, Fung CH. Quality indicators for the care of sleep disorders in vulnerable elders. Journal of the American Geriatrics Society. 2007;55:S424-30.

48. Zeng P, Meng B, Hong X, Liu XH. Recognition of mood disorders in elderly inpatients in a general hospital. Journal of the American Geriatrics Society. 2014;62(SUPPL. 2):S368.

49. Abreu W, Tolson D, Jackson GA, Staines H, Costa N. The relationship between frailty, functional dependence, and healthcare needs among community-dwelling people with moderate to severe dementia. Health & social care in the community. 2019;27(3):642-53.

50. Cao Y, Zhang W, Deng YT, Duan JX, Zhang M, Tu Q, et al. Risk factors associated with geriatric depression based on comprehensive geriatric assessment in chongqing area in China. Journal of the American Geriatrics Society. 2014;62(SUPPL. 2):S358.

51. Sugi T, Enomoto T, Ohara Y, Furuya K, Kitaguchi D, Moue S, et al. Risk factors for postoperative delirium in elderly patients undergoing gastroenterological surgery: A single-center retrospective study. Annals of Gastroenterological Surgery. 2023;7(5):832EP-40.

52. Blanquicett C, Cohen JB, Flowers C, Johnson T. The role of the comprehensive geriatric assessment in the evaluation of the older cancer patient. ONCOLOGY (United States). 2019;33(11):458EP-66.

53. Mc Carthy CE, O'Malley K, Geoghegan J, Mannion E, Costello M, Murphy R, et al. Screening for sleep disturbance as part of the comprehensive geriatric assessment. Age and Ageing. 2021;50(SUPPL 3).

54. Ageez D, El-Sobky H, Mohammad N, Rasheedy D, Shaltoot H. Self-efficacy and Optimism in Frail Older Men Without Functional Disability Attending Geriatric Outpatient Clinic: A Case-control Study. European Journal of Geriatrics and Gerontology. 2024;6(2):91EP-8.

55. Cheng H, Gurland BJ, Maurer MS. Self-reported lack of energy (anergia) among elders in a multiethnic community. The journals of gerontology Series A, Biological sciences and medical sciences. 2008;63(7):707-14.

56. Yu X, Wang D, Yao L, Shi Z, Liu X, Wu H, et al. Sleep disorders and the association with frailty among community-dwelling older adults in Northwest China: a cross-sectional study. BMJ open. 2025;15(2):e088030.

57. Ozkahraman S, Yildirim B, Ozkutuk N. Sleep disorders in elderly people : A literature review. Turk Geriatri Dergisi. 2010;13(SPEC. ISS. 1):50.

58. Cole C, Richards K. Sleep disruption in older adults: Harmful and by no means inevitable, it should be assessed for and treated. American Journal of Nursing. 2007;107(5):40EP-9.

59. Amato L, Giannetta N, Taborri S, Dionisi S, Panattoni N, Di Simone E, et al. Sleep Quality and Medication Adherence in Older Adults: A Systematic Review. CLOCKS & SLEEP. 2024;6(3):488-98.

60. Colon-Emeric CS. A team approach: comprehensive evaluation for functional decline in older patients. American family physician. 2013;88(6):397.

61. Rao A, Kumar S, Dhekale R, Krishnamurthy J, Mahajan S, Daptardar A, et al. Timed Up and Go as a predictor of mortality in older Indian patients with cancer: An observational study. Cancer Research, Statistics, and Treatment. 2022;5(1):75EP-82.

62. Try this: best practices in nursing care to older adults. The Pittsburgh Sleep Quality Index (PSQI)...Caregiver Strain Index (CSI). SCI Nursing. 2006;23(3):4p-p.

## Appendix 5: Preferred Reporting Items for Systematic reviews and Meta-Analyses extension for Scoping Reviews (PRISMA-ScR) Checklist

| **SECTION** | **ITEM** | **PRISMA-ScR CHECKLIST ITEM** | **REPORTED ON PAGE #** |
| --- | --- | --- | --- |
| **TITLE** | | | |
| Title | 1 | Identify the report as a scoping review. | 1 |
| **ABSTRACT** | | | |
| Structured summary | 2 | Provide a structured summary that includes (as applicable): background, objectives, eligibility criteria, sources of evidence, charting methods, results, and conclusions that relate to the review questions and objectives. | 3-4 |
| **INTRODUCTION** | | | |
| Rationale | 3 | Describe the rationale for the review in the context of what is already known. Explain why the review questions/objectives lend themselves to a scoping review approach. | 5-6 |
| Objectives | 4 | Provide an explicit statement of the questions and objectives being addressed with reference to their key elements (e.g., population or participants, concepts, and context) or other relevant key elements used to conceptualize the review questions and/or objectives. | 6 |
| **METHODS** | | | |
| Protocol and registration | 5 | Indicate whether a review protocol exists; state if and where it can be accessed (e.g., a Web address); and if available, provide registration information, including the registration number. | 6 |
| Eligibility criteria | 6 | Specify characteristics of the sources of evidence used as eligibility criteria (e.g., years considered, language, and publication status), and provide a rationale. | 7 |
| Information sources* | 7 | Describe all information sources in the search (e.g., databases with dates of coverage and contact with authors to identify additional sources), as well as the date the most recent search was executed. | 7 |
| Search | 8 | Present the full electronic search strategy for at least 1 database, including any limits used, such that it could be repeated. | Appendix 1 |
| Selection of sources of evidence† | 9 | State the process for selecting sources of evidence (i.e., screening and eligibility) included in the scoping review. | 7 |
| Data charting process‡ | 10 | Describe the methods of charting data from the included sources of evidence (e.g., calibrated forms or forms that have been tested by the team before their use, and whether data charting was done independently or in duplicate) and any processes for obtaining and confirming data from investigators. | 8 |
| Data items | 11 | List and define all variables for which data were sought and any assumptions and simplifications made. | 8 |
| Critical appraisal of individual sources of evidence§ | 12 | If done, provide a rationale for conducting a critical appraisal of included sources of evidence; describe the methods used and how this information was used in any data synthesis (if appropriate). | N/A |
| Synthesis of results | 13 | Describe the methods of handling and summarizing the data that were charted. | 9 |
| **RESULTS** | | | |
| Selection of sources of evidence | 14 | Give numbers of sources of evidence screened, assessed for eligibility, and included in the review, with reasons for exclusions at each stage, ideally using a flow diagram. | 9-10 |
| Characteristics of sources of evidence | 15 | For each source of evidence, present characteristics for which data were charted and provide the citations. | 11 |
| Critical appraisal within sources of evidence | 16 | If done, present data on critical appraisal of included sources of evidence (see item 12). | N/A |
| Results of individual sources of evidence | 17 | For each included source of evidence, present the relevant data that were charted that relate to the review questions and objectives. | 11-17 |
| Synthesis of results | 18 | Summarize and/or present the charting results as they relate to the review questions and objectives. | 11-17 |
| **DISCUSSION** | | | |
| Summary of evidence | 19 | Summarize the main results (including an overview of concepts, themes, and types of evidence available), link to the review questions and objectives, and consider the relevance to key groups. | 17-20 |
| Limitations | 20 | Discuss the limitations of the scoping review process. | 20-21 |
| Conclusions | 21 | Provide a general interpretation of the results with respect to the review questions and objectives, as well as potential implications and/or next steps. | 21 |
| **FUNDING** | | | |
| Funding | 22 | Describe sources of funding for the included sources of evidence, as well as sources of funding for the scoping review. Describe the role of the funders of the scoping review. | 21 |

JBI = Joanna Briggs Institute; PRISMA-ScR = Preferred Reporting Items for Systematic reviews and Meta-Analyses extension for Scoping Reviews.

* Where *sources of evidence* (see second footnote) are compiled from, such as bibliographic databases, social media platforms, and Web sites.

† A more inclusive/heterogeneous term used to account for the different types of evidence or data sources (e.g., quantitative and/or qualitative research, expert opinion, and policy documents) that may be eligible in a scoping review as opposed to only studies. This is not to be confused with *information sources* (see first footnote).

‡ The frameworks by Arksey and O’Malley (6) and Levac and colleagues (7) and the JBI guidance (4, 5) refer to the process of data extraction in a scoping review as data charting*.*

§ The process of systematically examining research evidence to assess its validity, results, and relevance before using it to inform a decision. This term is used for items 12 and 19 instead of "risk of bias" (which is more applicable to systematic reviews of interventions) to include and acknowledge the various sources of evidence that may be used in a scoping review (e.g., quantitative and/or qualitative research, expert opinion, and policy document).

*From:* Tricco AC, Lillie E, Zarin W, O'Brien KK, Colquhoun H, Levac D, et al. PRISMA Extension for Scoping Reviews (PRISMAScR): Checklist and Explanation. Ann Intern Med. 2018;169:467–473. [doi: 10.7326/M18-0850](http://annals.org/aim/fullarticle/2700389/prisma-extension-scoping-reviews-prisma-scr-checklist-explanation).
